# Structure–Activity Relationship Analysis of Immunoassay Systems for (Fluoro)quinolones Detection

**DOI:** 10.3390/ijms27188140

**Published:** 2026-09-12

**Authors:** Platon P. Chebotaev, Andrey A. Buglak, Nadezhda A. Byzova, Anatoly V. Zherdev, Olga D. Hendrickson

**Affiliations:** 1 Department of Molecular Biophysics and Polymer Physics, St. Petersburg State University, 7/9 Universitetskaya Embankment, 199034 St. Petersburg, Russia; st068500@student.spbu.ru; 2 Department of Quantum Electronics and Radiospectroscopy, Institute of Physics, Kazan Federal University, Kremlevskaya str. 18, 420008 Kazan, Russia; 3 A.N. Bach Institute of Biochemistry, Research Center of Biotechnology, Russian Academy of Sciences, Leninsky Prospect 33, 119071 Moscow, Russia; nbyzova@inbi.ras.ru (N.A.B.); zherdev@inbi.ras.ru (A.V.Z.); odhendrick@gmail.com (O.D.H.)

**Keywords:** antibiotics, fluoroquinolones, ofloxacin, ELISA, machine learning, binary classifications

## Abstract

Detection systems for antibiotics of the (fluoro)quinolone (FQ) group are in high demand among food safety regulators and agricultural inspectors. At the same time, the application of developed test systems may be complicated by the significant structural diversity of related FQs (including enantiomeric forms), which necessitates the careful evaluation of their selectivity. In this study, five polyclonal antibodies were generated by immunizing rabbits with the S- and R-enantiomers of ofloxacin (OFL) and its racemic mixture used as haptens. The cross-reactivity (CR) of the obtained antibodies toward 26 FQs, structural analogs of OFL, was evaluated using an indirect competitive enzyme-linked immunosorbent assay. Structure–activity relationship models were developed to analyze the obtained CR data. Three machine learning (ML) methods were applied: random forest classifier (RFC), logistic regression (LR), and a support vector classifier (SVC). The SVC model demonstrated the highest predictive performance in terms of the Log Loss metric. In contrast, the LR models showed the best overall balance across six statistical metrics, including precision, recall, and F1 score. Three-dimensional topological descriptors enabled discrimination between the S- and R-isomers of OFL, whereas constitutional and two-dimensional descriptors were less effective. The obtained results contribute to the development of FQ immunoassay systems and their computational analysis using ML approaches.

## 1. Introduction

**Figure 1:**
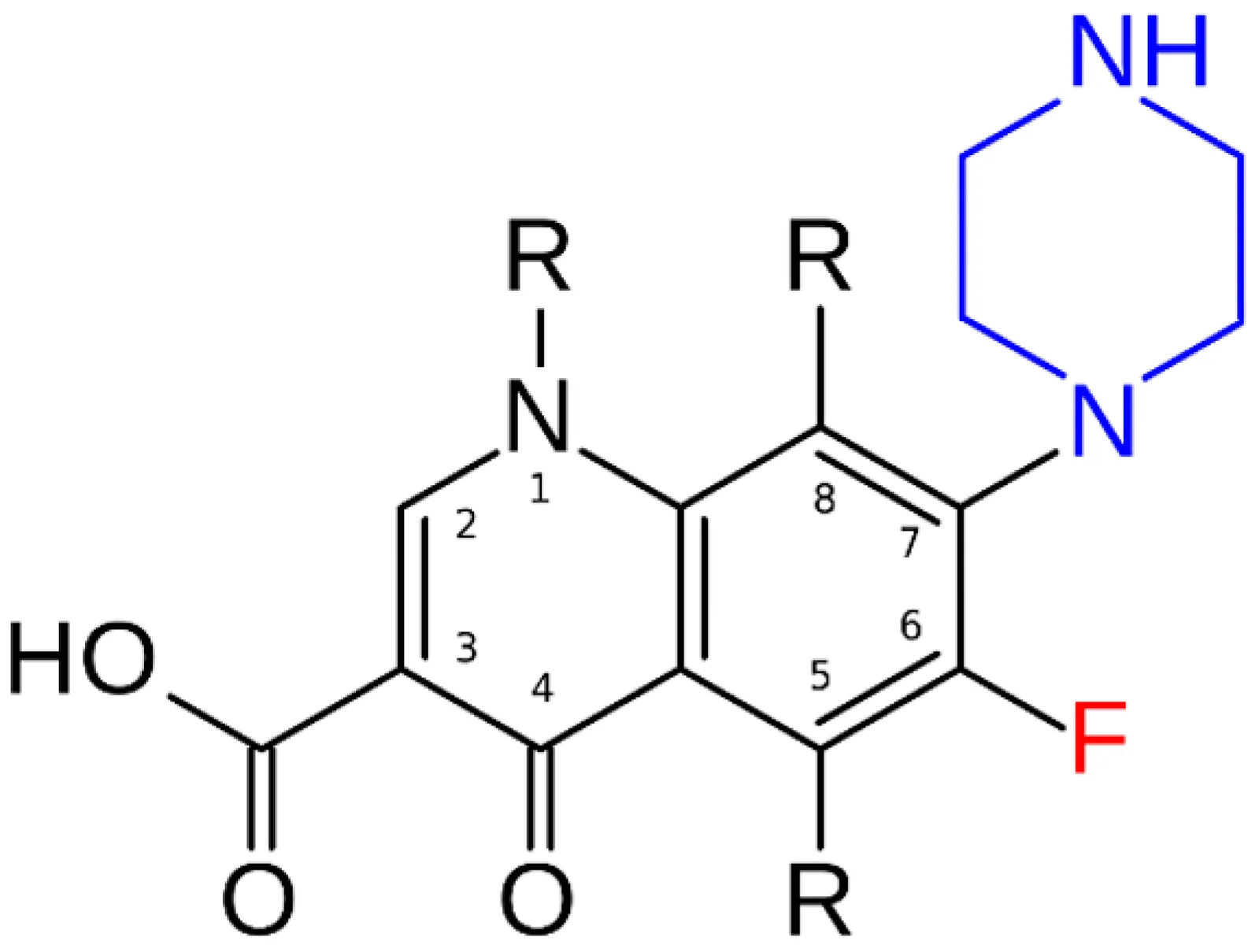
Parent structure of fluoroquinolone antibiotics with atom numbering.

(Fluoro)quinolones (FQs) are a class of synthetic antibiotics widely used in animal husbandry for the treatment and prevention of livestock infections [1,2,3]. The FQ group comprises several hundred compounds that differ in chemical structure and biological activity. Four generations of quinolones are generally distinguished. The antimicrobial activity of non-fluorinated quinolones of the first generation (e.g., nalidixic, oxolinic, and pipemidic acids) is primarily limited to Gram-negative bacteria. In contrast, fourth-generation FQs (e.g., moxifloxacin, gemifloxacin, and gatifloxacin) are modern broad-spectrum antibiotics used to treat severe infections caused by Gram-positive, Gram-negative, and anaerobic bacteria [4]. The core structure of FQs is based on a quinoline (or quinolone) scaffold—an aromatic nitrogen-containing heterocycle—responsible for their biological activity (Figure 1). A fluorine atom, typically located at position C6 of the quinolone ring, enhances the antimicrobial activity of FQs [5]. The carboxyl group at position C3 is essential for binding to the target enzymes of pathogenic bacteria, namely DNA gyrase and topoisomerase IV. Additional functional groups, including various aliphatic or aromatic substituents, may further modulate pharmacokinetic properties and the antibacterial spectrum.

Stereoisomerism of FQ molecules plays an important role in their biological activity [6,7]. Some FQs exist as enantiomeric pairs: mirror-image molecules with different spatial configurations. A key difference between enantiomers lies in their interactions with biological targets and, consequently, in their antimicrobial activity. In many cases, the levorotatory (S)-enantiomer exhibits greater antibacterial activity, whereas the dextrorotatory (R)-enantiomer may be less active or even inactive against certain microorganisms. Enantiomers may also differ in their pharmacokinetic properties (e.g., absorption, metabolism, and elimination rates), which influence the duration and intensity of drug action in the body. In addition, stereoisomers may differ in their safety profiles and side effects [8].

Ofloxacin (OFL) is a representative fluoroquinolone that exists as an enantiomeric pair due to the presence of a chiral center in its structure. The S-enantiomer of OFL is primarily responsible for antibacterial activity, whereas the R-enantiomer is significantly less active; S-OFL has been reported to be 8–128 times more active than the R-form, depending on the microorganism [9]. In clinical practice, OFL is often administered as a racemate (a 1:1 mixture of the two enantiomers). However, the use of the pure S-enantiomer instead of the racemic form allows dose reduction, elimination of the inactive R-isomer, improved pharmacokinetic properties, and potentially a reduced risk of adverse effects. Therefore, S-OFL is generally preferred over racemic OFL in modern clinical practice.

The use of FQs in animal husbandry may result in the contamination of food products, posing potential risks to human health [10]. FQs may exert adverse effects by disrupting the normal microbiota, leading to gastrointestinal disorders, including diarrhea and dysbiosis, as well as opportunistic infections such as candidiasis. Allergic reactions may also occur. Furthermore, the excessive use of FQs contributes to the development of antimicrobial resistance [11]. These concerns have stimulated the development of various analytical methodologies for the detection of FQs in food products and environmental samples [12,13,14,15,16]. Among these approaches, the enzyme-linked immunosorbent assay (ELISA), based on specific antigen–antibody interactions, is widely employed for FQ detection [17,18,19]. ELISA offers several advantages, including high sensitivity (down to pico- and even femtogram-level analyte concentrations), the capability for high-throughput screening of multiple samples, relatively low cost, ease of automation, rapid analysis (typically completed within 1–3 h), and methodological simplicity. ELISA methods for the determination of OFL have been reported in several studies [20,21].

Over the past decade, machine learning (ML) and (quantitative) structure–activity relationship ((Q)SAR) approaches have been extensively developed for the analysis of various types of data, including those related to FQs. (Q)SAR is a computational modeling methodology that establishes relationships between chemical structure and biological, physicochemical, or toxicological activity. It enables the prediction of molecular properties based on experimental activity data and calculated molecular descriptors and serves as a powerful tool in drug discovery, environmental science, and toxicology by accelerating research and reducing the need for costly laboratory testing. Several studies have characterized FQ-detecting immunoassay systems. In particular, pipemidic acid, norfloxacin, pazufloxacin, ciprofloxacin, gatifloxacin, lomefloxacin, sarafloxacin, and garenoxacin have been used as immunizing haptens to obtain “I”, “P”, and “Φ”-type hapten conformations for the production of polyclonal antibodies. These conformations demonstrated high specificity and low cross-reactivity (CR), in contrast to the “Y” conformation, which resulted in antibodies with broader specificity and increased CR [22]. Ciprofloxacin and clinafloxacin were employed as haptens in the development of immunoassays and were further investigated using 3D-QSAR analysis [23]. Recent advances in (Q)SPR and ML models addressing various aspects of FQ activity have been summarized in a comprehensive review [24].

In this study, five types of rabbit polyclonal antibodies were generated. The immunizing haptens included the two enantiomers of OFL (S- and R-isomers) as well as their racemic mixture (S, R-OFL). In four hapten–protein conjugates, the protein carriers were directly modified with the hapten, whereas in one conjugate, a linker (γ-aminobutyric acid) was used. The CR of the obtained antisera toward a panel of FQs was evaluated using an indirect competitive ELISA. The resulting data were analyzed using structure–activity relationship approaches and machine learning (ML) models. Three ML methods were applied: random forest classifier (RFC), logistic regression (LR), and a support vector classifier (SVC). Several predictive ML models were developed, and their performance was evaluated together with an analysis of molecular descriptor contributions. The results demonstrate that ML enables accurate prediction of the specificity of polyclonal antibodies, even when applied to a relatively small dataset of 26 FQ molecules, and allows for discrimination between stereoisomers. To the best of our knowledge, such differentiation has not been previously reported.

## 2. Results and Discussion

### 2.1. Experimental Evaluation of Antibody Cross-Reactivity by Indirect Competitive ELISA

Before structure–activity modeling, the specificity profiles of the obtained polyclonal antibodies were experimentally evaluated using indirect competitive ELISA. To detect small FQ haptens, an indirect competitive ELISA was employed. In this assay, a hapten–protein conjugate is immobilized in the wells of a microplate. The sample containing the antibiotic and specific antibodies is then added, resulting in a competition between the free FQ in the sample and the FQ present in the immobilized conjugate. Detection is achieved via an enzyme label introduced through anti-species antibodies, which produces a colorimetric signal that can be measured photometrically. As a result of this competitive interaction, the analytical signal is inversely proportional to the analyte concentration. Five antisera were analyzed: AS1, AS2, AS3, AS4, and AS5; the CR of each antiserum was assessed toward a panel of structurally related FQs by comparing the *IC50* values obtained for the target analyte and each cross-reactant.

The ELISA results demonstrated that the antibody specificity strongly depended on the structure of the immunizing hapten and the carrier conjugation strategy. AS1 was generated by immunization with the S-OFL–STI conjugate. In the AS1 system, the lowest *IC50* value and the highest CRs were observed for S, R-OFL: 0.034 ng/mL and 262%, respectively (Table 1). The same CR value was recorded for RUF. In contrast, the S-enantiomer of OFL exhibited a lower CR, with an *IC50* of 0.089 ng/mL and a CR of 100%. The CR values of five FQs (PEF, CIN, MAR, GAR, and ENR) ranged from 0.2% to 31.8%, whereas the remaining 18 compounds exhibited extremely low activity and CRs. Six molecules (S, R-OFL, SPAR, ENO, MOX, DIF, and NAL) were assigned to the test set using a random split prior to the structure–activity computations.

AS2, produced using the racemic S, R-OFL–STI immunogen, showed the highest response to S, R-OFL (CR was 100%) and retained substantial recognition of S-OFL (83.3%), RUF (26.9%), MAR (12.1%), and GAR (7.7%) (Table 1). In contrast, most other tested FQs showed negligible CR.

For AS3 production, the S-enantiomer of OFL conjugated with BSA was used as the immunogen. In the AS3 system, eight out of 26 molecules exhibited non-zero CRs, with the highest value (100%) observed for S-OFL (Table 2) and lower but measurable recognition of RUF (20.4%), R-OFL (16.9%), MAR (15.4%), and GAR (7.2%). The CRs of ENR, DIF, and PEF were in the range from 0.65 to 1.24% (Table 2).

The R-enantiomer of OFL conjugated with BSA was used for the production of AS4. The highest CR values were observed for R-OFL and MAR, both reaching 100% (Table 2). High CR was also detected for S-OFL and GAR (CR values of 75%), and a moderate response was observed for RUF (30%). In contrast, PEF, ENR, DIF, and LOM showed only weak CR (below 0.5%), whereas all remaining tested compounds exhibited negligible responses below 0.01%.

AS5, generated using S-OFL conjugated to BSA via the GABA linker as an immunogen, showed the highest CR toward S-OFL, which was used as the reference compound (100%) (Table 2). Among the structurally related FQs, the strongest response was observed for RUF, with a CR of 34.3%. Lower but still measurable CRs were detected for R-OFL, MAR, and GAR (16.2%, 12.9%, and 9.2%, respectively). PEF, DIF, and ENR produced only weak responses, with CR values below 0.3%, whereas the remaining tested FQs were practically non-reactive.

Thus, the experimentally determined CR values served as the activity dataset for the subsequent structure–activity analysis and machine-learning-based classification of FQ recognition by the obtained antisera.

### 2.2. Structure–Activity Modeling of the AS1 System

Following dataset preprocessing, structure–activity evaluations were conducted. When evaluated without cross-validation (CV) using RFC, LR, and SVC, all metrics (Precision, Recall, F1, ROC-AUC, and PR-AUC) exhibited perfect scores (1.0). This is because the training and testing were performed on the same dataset, which likely led to model overfitting (Table 3).

However, the Log Loss values were lower for RFC and SVC (0.086 and 0.087, respectively) compared to LR (0.117). This discrepancy can be attributed to differences in probability estimation: the logistic regression model predicted intermediate probabilities in the range of 0.7–0.9, whereas the random forest and support vector classifier produced near-binary probabilities (0 or 1). As a result, despite identical classification outcomes, RFC and SVC yielded lower Log Loss values.

Upon CV, the statistical metrics became more moderate. The RFC model successfully captured positive instances (high Recall) but produced a relatively high number of false positives, resulting in lower Precision. LR demonstrated the best balance between Precision and Recall, correctly identifying almost all class “1” molecules while generating only a small number of false positives. The SVC produced better metrics than RFC but was slightly inferior to LR. Among the tested models, LR with CV (LR + CV) achieved the most balanced performance and was the most robust to data imbalances.

Analysis of the linear models (LR and SVC) revealed that:

• CATS2D_02_AL exerted an inverse effect on activity;
• nRNR2 and RDF130m were highly correlated with immunochemical activity;
• JGI5 was inversely proportional to CR in the AS1 models (Table 4).

The RFC model identified JGI5, RDF130m, and CATS2D_02_AL as the most important descriptors, which was generally consistent with the findings from the linear models (Figure S1).

The JGI5 descriptor was inversely correlated with CR. JGI5 evaluates charge transfer between pairs of atoms separated by a topological distance of five bonds. For FQs, this descriptor allows the electronic effects of substituents to be distinguished, reflecting how substituents at positions C7 (e.g., piperazine) and C8 influence both the electron density of the quinolone core and the overall molecular polarizability. Topological charge indices are known to correlate with the dipole moment and the ability of a molecule to engage in electrostatic interactions with biological targets. Among the analyzed compounds, NAL exhibited the highest JGI5 value (0.04201), likely due to the absence of a fluorine atom and the specific charge distribution in its simplified core structure. RUF had the lowest value (0.02778), which may be attributed to the presence of sulfur in the additional ring. S-OFL and S,R-OFL had identical values (0.03216), reflecting the identical topological distances between the relevant atoms. The JGI5 values are summarized in Table S2.

The nRNR2 descriptor (directly proportional to CR), which represents the number of tertiary amines, takes values of either 0 or 1 and thus functions as a binary classifier. Compounds with nRNR2 = 1 contain a fully substituted nitrogen atom in the side chain (e.g., substituted with a methyl or ethyl group). S-OFL and S, R-OFL feature a methylated nitrogen in the oxazine ring or side chain, whereas ENR and PEF possess an ethyl- or methyl-substituted piperazinyl moiety. Molecules with nRNR2 = 0 either contain a secondary amino group (with a free hydrogen atom) or lack such groups entirely. Examples include CIP, NOR, and MOX, which have unsubstituted piperazine or other rings with a secondary nitrogen. In GAT, LOM, and SPAR, the nitrogen atom present in the structure is not tertiary.

The CATS2D_02_AL descriptor is a 2D topological descriptor that measures the number of atomic pairs in a molecule in which one atom is a H-bond acceptor and the other is located in a lipophilic region. The values in Table S2 range from 6 to 14. A low number of pairs (6–7) is observed for MAR, RUF, CIN, OFL, and LOM, while a medium number of pairs (8–10) is observed for GAT, ORB, ENO, PAZ, CIP, and NAL. ENR (11), DAN (12), and NAD (14) exhibit the highest values of this descriptor. CATS2D_02_AL differentiates molecules according to their local structural features, in which the acceptor and lipophilic moiety are positioned close to each other (e.g., the nature of the side chain at C7, such as piperazine, oxazine, or cyclopropyl), since these groups determine the presence and relative location of both H-bond acceptors and lipophilic regions. NAL and CIN exhibit intermediate values, whereas some modern FQs (DAN and NAD) display higher values, reflecting a more complex molecular structure enriched with functional groups.

RDF130m is a complex 3D descriptor used to characterize molecular structure. It is based on the radial distribution function, a concept from statistical mechanics that describes the probability of finding an atom at a certain distance from another atom. RDF130m measures the atomic density distribution as a function of distance from a central atom or the center of mass, specifically at a distance of 13 Å, with the contribution of each atom weighted by its atomic mass. This descriptor captures subtle differences in the 3D shape, size, and mass distribution of molecules. Its values vary widely, from 0 to 3.7. High values (e.g., GAR, 3.738) indicate the presence of bulky substituents or an extended molecular conformation. RDF130m also distinguishes between S-OFL (1.245) and S,R-OFL (1.344), whereas zero values (e.g., CIP, DAN, ENO) reflect more compact or symmetrical structures.

E_Total_ is an indicator of molecular size. CLI exhibits the lowest total energy (−1607.5 a.u.), as it is one of the largest and heaviest molecules in the dataset, whereas NAL has the highest total energy (−799.4 a.u.), reflecting its status as the smallest and lightest structure.

VE1_D/Dt is a 2D topological descriptor derived from the distance/detour matrix. It corresponds to the sum of the coefficients of the last eigenvector of the matrix and characterizes molecular topology by extracting information from either the distance matrix (shortest paths between all pairs of atoms) or the detour matrix (longest paths without repeating vertices). The VE1_D/Dt values range from 3.457 (NAL) to 4.905 (MOX). High values, such as those for MOX and GAR, indicate more complex and branched structures, particularly in the side chains, whereas low values, as observed for NAL and CIN, correspond to simpler, less functionalized molecules. The VE1_D/Dt values for S-OFL and S, R-OFL are identical (3.862), confirming that this descriptor is purely 2D topological and does not capture 3D chirality.

### 2.3. Structure–Activity Modeling of the AS2 System

The performance metrics of the ML models developed for the AS2 system are summarized in Table 5.

For the RFC and SVC models of AS2, inflated metrics (all equal to 1.0) were observed on the training set, indicating overfitting. In contrast, logistic regression exhibited a Precision of 0.5, a Recall of 1.0, and an elevated Log Loss of 0.271. To obtain an objective assessment of model performance, a stratified repeated CV was applied, and LR + CV achieved the best balance among the evaluated metrics.

With CV, the results provide a more realistic assessment of model performance. RFC exhibits high sensitivity (Recall), but this comes at the cost of an increased number of false positives, resulting in lower Precision. LR achieves the most balanced performance, reliably identifying nearly all positive samples while generating fewer false positives. SVC performs better than RFC but slightly underperforms compared to LR, although its performance remains relatively stable. Overall, LR + CV provides the optimal combination of metrics and demonstrates the greatest robustness to class imbalance.

Regarding descriptor contributions, the linear models indicate that VE1_D/Dt negatively influences activity prediction (Table 6), reflecting an inverse relationship between its value and the probability of the positive class. In contrast, CATS3D_11_AA and qpmax consistently exhibit a positive impact across all models, highlighting their key role in determining immunochemical activity. Additional contributions are provided by the topological descriptor PW4 and the spatial descriptor RDF105v, which are particularly important in the RFC and SVC models. Overall, the AS2 models suggest that a combination of spatial descriptors (CATS3D, RDF105v), topological descriptors (PW4, VE1_D/Dt), and molecular charge characteristics (qpmax) constitutes the primary factors governing activity in the AS2 system.

Molecular descriptor values used by the AS2 models are listed in Table S3. The CATS3D_11_AA descriptor takes values of either 2 (seven molecules) or 0 (19 molecules), yet it contributes significantly to the SVC model (34.9%) (Figure S2). In the AS2 models (and also in AS3 and AS5 models), the presence of this descriptor reflects two key aspects:

• Specific pharmacophore pairs: CATS3D_11_AA identifies whether a specific spatial pair of H-bond acceptors is present within the molecule.
• 3D conformation: The descriptor captures how these functional groups are arranged relative to each other in space.

Specifically, in the AS2 models, CATS3D_11_AA represents the presence of a carboxyl group (H-bond acceptor) and an N4′-substituted piperazine ring (second H-bond acceptor at the N4′ atom). Molecules lacking a piperazine ring at the C7 position, or possessing a hydrogen atom at N4′, exhibit a CATS3D_11_AA value of 0. In contrast, S,R-OFL, MAR, RUF, PEF, ENR, and DIF contain two H-bond acceptors positioned at a specific distance within their 3D conformation. Molecules such as GAR, LOM, CIN, and MOX either lack a second acceptor pair at the required distance, or their spatial arrangement does not satisfy the criteria for this descriptor.

The Qpmax descriptor provides insight into the most electrophilic region of a molecule, typically corresponding to the site most likely to interact with an electron-rich biological target or to undergo nucleophilic reactions. Among the analyzed compounds, GAR exhibits the highest maximum positive partial charge (0.3893), indicating the most electrophilic center, followed by CIN (0.3609). Most other FQs, including CIP, MOX, and OFL, share an identical Qpmax value of 0.342, suggesting a consistent localization of positive charge in their core structure regardless of variations in their side chains. The Qpmax descriptor therefore allows differentiation of the electronic properties of the core structure, highlighting regions most susceptible to chemical attack or interaction.

PW4 is a 2D topological descriptor that quantifies molecular branching and size based on the number and arrangement of paths of length four within the molecular graph. The PW4 values for FQs generally fall within a narrow range (approximately 0.185–0.211). MAR, OFL, and NAD share an identical value of 0.2044, indicating equivalent 2D topological connectivity for four-atom paths in their core structures. MOX exhibits one of the highest values (0.2107), reflecting a more highly branched or complex structure compared to compounds such as ENR and DIF (both 0.1854). PW4 does not distinguish between the R- and S-isomers of OFL, as both display the same value of 0.2044.

RDF105v (the 10.5 Å volume-weighted van der Waals radial distribution function) is a 3D descriptor that characterizes the spatial distribution of atoms in FQs. The descriptor values range from 0 to 0.5 for DAN, FLU, OXO, and NAL; from 0.6 to 2.0 for S-OFL, ENO, GAT, LOM, and CIP; and from 2.1 to above 4.0 for GAR, NAD, PEF, ORB, and DIF. High RDF105v values indicate the presence of extended 3D structures in which many atoms are positioned at approximately 10.5 Å from each other. RDF105v clearly distinguishes between S-OFL (0.6824) and S, R-OFL (1.1417), demonstrating its sensitivity to 3D stereochemistry. Zero or very low values, as observed for DAN and FLU, likely reflect more symmetrical or compact structures where atoms are not positioned at the specified distance.

MDEN-23, another topological descriptor, equals 0 for 13 molecules, while PIP exhibits the highest value (4.17). This 2D index quantifies the topological distances between all pairs of secondary and tertiary nitrogen atoms in a molecule by summing or averaging the shortest bond path lengths between each possible pair. The MDEN-23 values presented in Table S3 are discrete and include 0, 0.125, 0.4364, 0.5, 1, 1.321, and 2.027. Molecules such as S, R-OFL, NAD, MOX, DAN, CLI, DIF, PAZ, FLU, OXO, NAL, CIN, PEF, ENR, RUF, and MAR either lack secondary and tertiary nitrogen atoms entirely or have them arranged such that the descriptor equals 0. CIP, GAT, ORB, LOM, SPAR, and SAR (0.4364) contain at least one pair of secondary and tertiary nitrogen atoms at specific topological distances. ENO (1.321) and PIP (2.027) exhibit more complex arrangements of >NH- and -N< pairs or longer distances between them. Overall, MDEN-23 provides a quantitative measure of how far key nitrogen-containing functional groups are separated within the molecular graph. Next, we shall analyze the BSA-based immunogen systems.

### 2.4. Structure–Activity Modeling of the AS3 System

Six compounds—R-OFL, ORB, SPAR, TOS, CIP, and NAL—were randomly assigned to the test set prior to structure–activity relationship modeling. The statistical metrics for the AS3 system are summarized in Table 7.

Without CV, all three models (RF, LR, and SVC) yield perfect class metrics (Precision, Recall, F1, ROC-AUC, PR-AUC = 1.0), indicating overfitting on the training set. However, the Log Loss values differ: SVC exhibits the lowest value (0.028), LR shows a moderate value (0.084), and RFC has the highest value (0.143). These differences arise from the way each model treats predicted probabilities: SVC produces nearly binary probabilities (close to 0 or 1), LR generates smoother probability estimates, and RFC demonstrates the least precise probability calibration on the training data.

Logistic regression with CV demonstrates the best balance between precision and recall. This model reliably identifies positive samples while producing a minimal number of false positives, as reflected by its F1 score of 0.935 ± 0.163—the highest among the AS3 models. In comparison, SVC with CV achieves a slightly lower F1 score (0.912 ± 0.185) but outperforms logistic regression in terms of Log Loss, indicating more accurate probability estimates. Therefore, logistic regression is preferable when an optimal trade-off between precision and recall is desired, whereas the SVC model is more suitable for tasks requiring stable and confident probability predictions.

The RFC model identifies Eig05_EA(dm), CATS3D_11_AA, R3m, Mor24v, E_LUMO_, and SsssCH as the most significant features (Figure S3), which generally aligns with the results from the linear models. In the linear models, CATS3D_11_AA positively influences the probability of class “1”, whereas R3m, E_LUMO_ (eV), and SsssCH are inversely correlated with the CR value (Table 8).

Eig05_EA(dm) demonstrates the highest feature importance across the AS3–AS5 system models, contributing negatively in logistic regression (−17.5%) and positively in SVC (35.0%). The highest Eig05_EA(dm) values are observed for R-OFL, S-OFL, and RUF (0.8), while 19 molecules possess a zero value (Table S4). For NAD, a zero value reflects a rigid, compact, and highly annelated structure, resulting in a simpler molecular graph. In contrast, the high value for OFL (0.8) indicates greater structural flexibility and branching, influenced by the presence of a free piperazinyl group and a dangling oxazine ring that increase the complexity of the molecular graph. Interestingly, Eig05_EA(dm) does not distinguish between the R- and S-isomers of OFL.

The R3m descriptor can distinguish between R-OFL (0.6644) and S-OFL (0.6439). Using 3D coordinates, R3m captures differences in the spatial arrangement of chiral centers. The wide range of R3m values reflects how the 3D shape of FQs varies depending on their substituents, which directly influences their ability to bind biological targets. The zero value observed for danofloxacin likely results from its molecular symmetry, which causes R3m to be set to zero during calculation.

Mor24v values range from negative (−0.2686 for MOX) to positive (0.434 for GAR), highlighting the diversity of 3D structures among FQs. A low Mor24v value, as in MOX, likely indicates a specific volume distribution and symmetry, whereas NAD (−0.0102) reflects a compact and rigid structure. GAR exhibits the highest Mor24v value in the dataset (0.434), indicating either a large effective van der Waals volume or pronounced structural asymmetry. Similar to R3m, Mor24v also differentiates between R-OFL (0.0447) and S-OFL (0.2152).

E_LUMO_ energy is a quantum chemical descriptor that characterizes a molecule’s ability to accept electrons and is commonly used as a measure of electrophilicity or electron affinity. PIP and GAT have the lowest LUMO energies (−0.9288 eV and −0.8991 eV, respectively), indicating high electrophilicity, whereas SPAR exhibits the highest LUMO energy (−0.396 eV).

SsssCH is a 2D, conformation-independent topological descriptor belonging to the family of electro-topological state (E-state) indices. It is calculated based on atomic connectivity within the molecular graph and quantifies the combined electronic and structural influence of all tertiary carbon atoms (>CH-) in a molecule. For FQs, SsssCH characterizes the degree of branching and the presence of tertiary carbons, providing insight into the nature of aliphatic substituents. Descriptor values range from 0 (for simpler FQs such as NAL, CIN, and PEF) to 0.9186 for DAN. Low values indicate the absence of tertiary carbons in the core structure, whereas high values, such as for DAN (0.9186) and MOX (0.8874), reflect branched chains or complex substituents. Accordingly, SsssCH conveys how branching and tertiary carbon content influence physicochemical properties of FQs, including lipophilicity.

Other molecular descriptors used in the AS4 and AS5 system models are discussed in Section 2.5 and Section 2.6, respectively.

### 2.5. Structure–Activity Modeling of the AS4 System

Without CV, all models exhibit perfect performance (Precision, Recall, F1 score, ROC-AUC, PR-AUC = 1.0), indicating overfitting, as testing was performed on the same dataset (Table 9). However, Log Loss values differ: RFC and SVC show lower values (0.056–0.058) compared to LR (0.113), reflecting the more “rigid” probabilistic predictions produced by decision trees and SVC. With CV, the metrics become more realistic. RFC demonstrates high sensitivity (Recall = 0.96), meaning it reliably identifies almost all positive samples, but its Precision is lower (0.83) due to false positive predictions. LR and SVC exhibit more balanced behavior: LR + CV achieves the highest F1 score (0.953) and perfect Recall (1.0), with minimal loss in Precision, while SVC + CV remains stable, yielding a slightly lower F1 score (0.943) but outperforming LR in Log Loss (0.104 vs. 0.152), indicating more confident probabilistic predictions.

The Eig05_EA(dm) descriptor (Table S5) contributes most significantly to class separation, showing a positive correlation with activity and maintaining similar importance across all three models. In contrast, the VE2_D/Dt descriptor negatively impacts activity, reducing the probability of belonging to class “1”. SsssCH, which reflects the number of tertiary carbon atoms, exhibits a comparable negative effect, whereas the N-068 descriptor contributes positively to activity. Spatial pharmacophore interactions captured by CATS3D_03_AN, along with global 3D molecular properties reflected in Mor24u, show moderate but consistent significance. The RFC model, however, identifies VE2_D/Dt as the most influential descriptor, in general agreement with the findings from the linear models (Table 10, Figure S4).

The N-068 descriptor denotes the number of hydroxyl groups attached to aromatic rings (Ar–OH). Values of N-068 range from 0 to 2. OFL contains one Ar–OH group, whereas MAR has two. The presence of these groups increases molecular polarity and the capacity for hydrogen bonding, thereby affecting solubility and interactions with biological targets. Most other FQs, such as CIP, MOX, and LOM, lack aromatic hydroxyl groups (N-068 = 0), generally making them more lipophilic.

CATS3D_03_AN differentiates FQs based on their 3D conformation and charge distribution. This descriptor takes discrete values of 0, 1, or 2. For DAN (CATS3D_03_AN = 0), no acceptor–negative charge pair exists at a distance of 3 Å. Most FQs, including CIP, MOX, and S-OFL, possess one such pair in the optimal 3D conformation. R-OFL and MAR exhibit two acceptor–negative pharmacophore pairs simultaneously. Accordingly, CATS3D_03_AN successfully distinguishes between S-OFL (1) and R-OFL (2), reflecting subtle differences in the spatial orientation of chiral centers.

Mor24u values range from −0.6387 (MOX) to 0.3441 (GAR), highlighting the diversity of 3D molecular structures. Low or negative values (−0.6387 to −0.19) are observed for MOX, NAD, PIP, ENO, and RUF, while values from −0.18 to 0.10 occur for TOS, FLU, OXO, NAL, ORB, and CLI. Higher values (0.17–0.34) are found for S, R-OFL, SAR, GAT, ENR, and GAR. As a 3D descriptor, Mor24u captures subtle differences in molecular spatial organization that 2D descriptors cannot. It also differentiates S-OFL (0.1724) from R-OFL (0.0822), confirming its sensitivity to stereochemistry.

### 2.6. Structure–Activity Modeling of the AS5 System

Without CV, all models exhibit perfect performance (Precision, Recall, F1-score, ROC-AUC, PR-AUC = 1.0), reflecting overfitting (Table 11). Differences are apparent only in the Log Loss values: 0.104 for RF, 0.076 for SVC, and 0.094 for LR.

With CV, the metrics become more realistic. RFC demonstrates high sensitivity (Recall = 0.700) but suffers in Precision (0.545), indicating a substantial number of false positives. Logistic regression (LR + CV) achieves a balanced performance (F1 = 0.900, Recall = 0.930, Precision = 0.888), providing the most robust prediction of activity. SVC + CV also produces reasonably good results (Recall = 0.830, Precision = 0.725) but is generally inferior to LR + CV in terms of F1-score and shows higher Log Loss (0.185 vs. 0.166 for LR + CV). Overall, among cross-validated models, LR + CV demonstrates the best performance across all metrics.

Interpretation of the descriptors indicates that Eig05_EA(dm) is the leading predictor of activity (Figure S5), being equally important across all three models and positively correlated with immunochemical activity (Table S6). The N-068 descriptor, representing the number of aromatic hydroxyl (Ar–OH) groups, also makes a significant contribution, increasing the probability of belonging to the active class. In contrast, SsssCH exhibits a negative effect, reducing the likelihood of positive activity. Spatial descriptors, such as CATS3D_11_AA and RDF035v, play a secondary role but still contribute to class differentiation. C-006, an E-state index for –CH– groups, has a modest yet consistent influence.

Overall, these results suggest that activity in this system is primarily determined by the topological and electronic properties of the molecular graph (Eig05_EA(dm)) and the presence of hydroxyl substituents (N-068), while structural parameters—such as –CH– groups, pharmacophore pairs, and atomic distribution—fine-tune the predictions, refining the boundaries between classes.

The first four descriptors listed in Table 12 were discussed in previous sections. Therefore, RDF035v and C-006 were analyzed, which have not been examined earlier. High values of GAR (12.12) and GAT (10.1) indicate the presence of bulky structures or a specific extended conformation, where many atoms are located at precisely 3.5 Å. RDF035v clearly distinguishes S-OFL (4.016) and R-OFL (6.11), confirming its sensitivity to 3D stereochemistry, as it accounts for precise spatial coordinates and atomic volumes. Lower values for basic structures such as NAL (2.633) reflect their smaller size and simplicity compared to third- and fourth-generation FQs.

High values (C-006 = 5–6) are characteristic of FQ molecules with long/branched aliphatic chains or rings, such as OFL, RUF, or PEF. Low values (C-006 = 0–1) are characteristic of more compact/aromatic structures, such as FLU, NAL, or GAR. OFL isomers have the same C-006 value, confirming that it is a 2D descriptor, which is not sensitive to 3D chirality.

### 2.7. Limitations of the Study and Matrix Effects

In this study, polyclonal antibodies were used as recognition elements. Polyclonal antisera represent heterogeneous mixtures of immunoglobulins that may differ in affinity, specificity, and contribution to the overall immunochemical response. Therefore, the CR values obtained by ELISA should be interpreted as averaged assay-level responses rather than as binding characteristics of a single antibody clone. Such heterogeneity may introduce additional experimental noise, broaden the apparent specificity profile, and complicate the direct mechanistic interpretation of individual antibody–hapten interactions.

This limitation may be relevant for (Q)SAR modeling, because the activity endpoint used in this study reflects the cumulative competitive response of antibody populations toward each FQ. As a result, the developed models describe structure-dependent recognition patterns of complete immunoassay systems rather than isolated molecular interactions between a single antibody and antigen. Nevertheless, all antisera were evaluated under standardized ELISA conditions, and repeated stratified cross-validation was applied to reduce the risk of overinterpreting model performance on a small and imbalanced dataset. Future studies using monoclonal or recombinant antibodies will allow for more precise assessment of structure–recognition relationships and improve the reproducibility of structure–activity models for immunoassay design.

Another important aspect for practical application is the potential influence of matrix effects. In the present study, antibody CR was evaluated under standardized ELISA conditions, and the obtained values were used as the activity data for structure–activity relationship modeling. Therefore, the developed models characterize structure-dependent recognition patterns of the antibody systems rather than the complete analytical performance of the assays in complex samples. For food and environmental monitoring, matrix components may affect several stages of immunochemical analysis. In addition, sample preparation may influence analyte extraction efficiency and recovery, especially in complex food matrices. These factors may change the apparent sensitivity and specificity of the assay compared with buffer-based measurements. Thus, the present structure–activity models should be considered as a tool for predicting and interpreting antibody recognition of structurally related FQs under controlled immunochemical conditions. Their practical implementation for residue monitoring will require matrix-specific optimization, including appropriate sample pretreatment, dilution or extraction procedures, recovery studies, and comparison with reference analytical methods. Nevertheless, the CR profiles obtained in this study provide a useful basis for selecting antibody systems with suitable specificity for further development and validation of FQ immunoassays in real food and environmental samples.

## 3. Materials and Methods

### 3.1. Reagents and Materials

In this study, S-OFL from Santa Cruz Biotechnology (Santa Cruz, CA, USA) and R-OFL from MedChemExpress (Monmouth Junction, NJ, USA) were used. A racemic mixture of OFL (S, R-OFL), cinoxacin (CIN), ciprofloxacin (CIP), clinafloxacin (CLI), danofloxacin (DAN), difloxacin (DIF), enoxacin (ENO), enrofloxacin (ENR), flumequine (FLU), gatifloxacin (GAT), garenofloxacin (GAR), lomefloxacin (LOM), marbofloxacin (MAR), moxifloxacin (MOX), nadifloxacin (NAD), nalidixic acid (NAL), orbifloxacin (ORB), oxolinic acid (OXO), pazufloxacin (PAZ), pefloxacin (PEF), pipemidic acid (PIP), rufloxacin (RUF), sarafloxacin (SAR), sparfloxacin (SPAR), tosufloxacin (TOS), gamma-aminobutyric acid (GABA), 1-ethyl-3-(3-dimethylaminopropyl) carbodiimide (EDC), N,N′ -dicyclohexylcarbodiimide (DCC), N-hydroxysuccinimide (NHS), bovine serum albumin (BSA), soybean trypsin inhibitor (STI), ovalbumin (OVA), triethylamine, and dimethylformamide (DMF) from Sigma-Aldrich (St. Louis, MO, USA) were used. Goat anti-rabbit immunoglobulins (GARI) conjugated with horseradish peroxidase (GARI–HRP) were purchased from Arista Biologicals (Allentown, PA, USA). A ready-to-use TMB-based substrate solution was obtained from Immunotech (Moscow, Russia). All other chemicals were of analytical grade (Khimmed, Moscow, Russia). All solutions were prepared with ultrapure water with a resistivity of at least 18.2 MΩ × cm (at 25 °C) obtained on a Simplicity^®^ water purification system (Millipore Corporation, Burlington, MA, USA). For the enzyme-linked immunosorbent assay (ELISA), polystyrene 96-well transparent microplates from Costar (Corning, Tewksbury, MA, USA) were utilized.

### 3.2. Synthesis of Hapten–Protein Conjugates as Immunogens and a Coating Antigen

Six hapten–protein conjugates were synthesized using carbodiimide-mediated activation [21]: S-OFL–BSA, R-OFL–BSA, S-OFL-GABA–BSA, S-OFL-GABA–OVA, S-OFL–STI, and S, R-OFL–STI (the synthetic routes are schematically presented in Figure S6). Concentrations of the reactants during synthesis are indicated in Table S1. Haptens were dissolved in DMF (0.5 mL), and then the activators were added. The reaction mixtures were stirred at room temperature (RT) for 2 h. After that, if no linker was introduced between the hapten and the protein (conjugates S-OFL–BSA, R-OFL–BSA, S-OFL–STI, and S, R-OFL–STI), the activated hapten solution was added dropwise to the BSA or STI solutions in 50 mM sodium carbonate buffer, pH 9.5, and stirred for 2.5 h at RT and then overnight at 4 °C.

For S-OFL-GABA–BSA and S-OFL-GABA–OVA conjugates, S-OFL and activators were dissolved in DMF and stirred overnight at RT. After that, the GABA linker was added to the activated solution and incubated in the same conditions. Then, the carboxyl group of the GABA linker was activated. BSA or OVA was dissolved in the 50 mM sodium carbonate buffer, pH 9.5, and DMF (200 µL) was added dropwise to the protein solution. After that, the activated S-OFL-GABA solution was added dropwise to BSA or OVA solutions, and the reaction mixtures were stirred for 5 h at RT.

Although possible side reactions, including self-crosslinking of activated low-molecular-weight FQ derivatives, cannot be completely excluded under carbodiimide-mediated coupling conditions, the reaction mixtures were purified after conjugation. Insoluble by-products were removed by centrifugation at 5000× *g* for 10 min. The supernatants containing the hapten–protein conjugates were dialyzed against 50 mM phosphate-buffered saline containing 100 mM NaCl, pH 7.4, to remove low-molecular-weight by-products, unreacted haptens, linker molecules, residual coupling reagents, and organic solvent. The purified conjugates were stored at −18 °C until use.

### 3.3. Production of Polyclonal Antibodies

To produce polyclonal antibodies, the immunization protocol described in [21] was used. One Chinchilla rabbit was immunized with each hapten–protein conjugate, corresponding to five rabbits in total. The animals weighing 3–4 kg were immunized with S-OFL–STI, S, R-OFL–STI, S-OFL–BSA, R-OFL–BSA, and S-OFL-GABA–BSA conjugates at day 1 (subcutaneously, with complete Freund’s adjuvant, 1:1 *v*/*v*, 1 mg/rabbit by protein) and at days 22, 36, 50, and 64 (subcutaneously, with incomplete Freund’s adjuvant, 1:1 *v*/*v*, 0.5 mg/rabbit by protein). At days 44, 57, and 72, blood was collected from the ear vein. The antisera were separated, divided into aliquots of 500 µL, and stored at −20 °C. For further experiments, antisera collected at day 44 were used, hereafter called AS1 (immunogen–S-OFL–STI), AS2 (immunogen–S, R-OFL–STI), AS3 (immunogen–S-OFL–BSA), AS4 (immunogen–R-OFL–BSA), and AS5 (immunogen–S-OFL-GABA–BSA).

### 3.4. Assessment of Antibody Specificity by the Indirect Competitive ELISA

To study the specificities of AS1 and AS2, S-OFL-GABA–BSA conjugate (0.9 µg/mL, 100 µL in PBS) was immobilized in the microplate wells overnight at 4 °C. To study the specificities of AS3, AS4, and AS5, the S–OFL–GABA–OVA conjugate was adsorbed under the same conditions. After that, the microplate was washed four times with PBS containing 0.05% Triton X-100 (PBST). Then, solutions of FQs (S-OFL, R-OFL, S, R-OFL, GAR, MAR, RUF, CIN, PEF, ENR, GAT, ORB, LOM, SPAR, ENO, NAD, TOS, MOX, DAN, CLI, CIP, DIF, PAZ, SAR, FLU, PIP, NAL, and OXO) (1 µg/mL–0.1 pg/mL, 50 µL in PBST) and AS (in dilutions of 1:50,000 for AS1, 1:30,000 for AS2, 1:100,000 for AS3, 1:300,000 for AS4, and 1:50,000 for AS5, 50 µL in PBST) were added to the wells and incubated for 1 h at 37 °C. After washing the microplate as described above, GARI–HRP (in dilution of 1:5000, 100 µL in PBST) was added to the wells and incubated for 1 h at 37 °C. After washing, the HRP activity was measured. For this, a TMB solution (100 µL) was added to the microplate wells and incubated for 10–15 min at RT. The reaction was terminated by adding 1 M sulfuric acid (50 µL), and the optical density was measured at 450 nm (OD_450_) on a Zenyth 3100 microplate spectrophotometer (Anthos Labtec Instruments, Wals, Austria). Based on the obtained concentration–response curves, CR values were assessed as follows:

(1) CR = *IC50*_targetFQ_/*IC50*_cross-reactant_ × 100%

where *IC50* is the analyte concentration resulting in 50% inhibition of the detected colorimetric signal. All *IC50* values were calculated from three replicate ELISA measurements (n = 3) and are presented as mean ± standard deviation (SD). CR values were calculated using the mean *IC50* values.

### 3.5. Preprocessing

**Figure 2:**
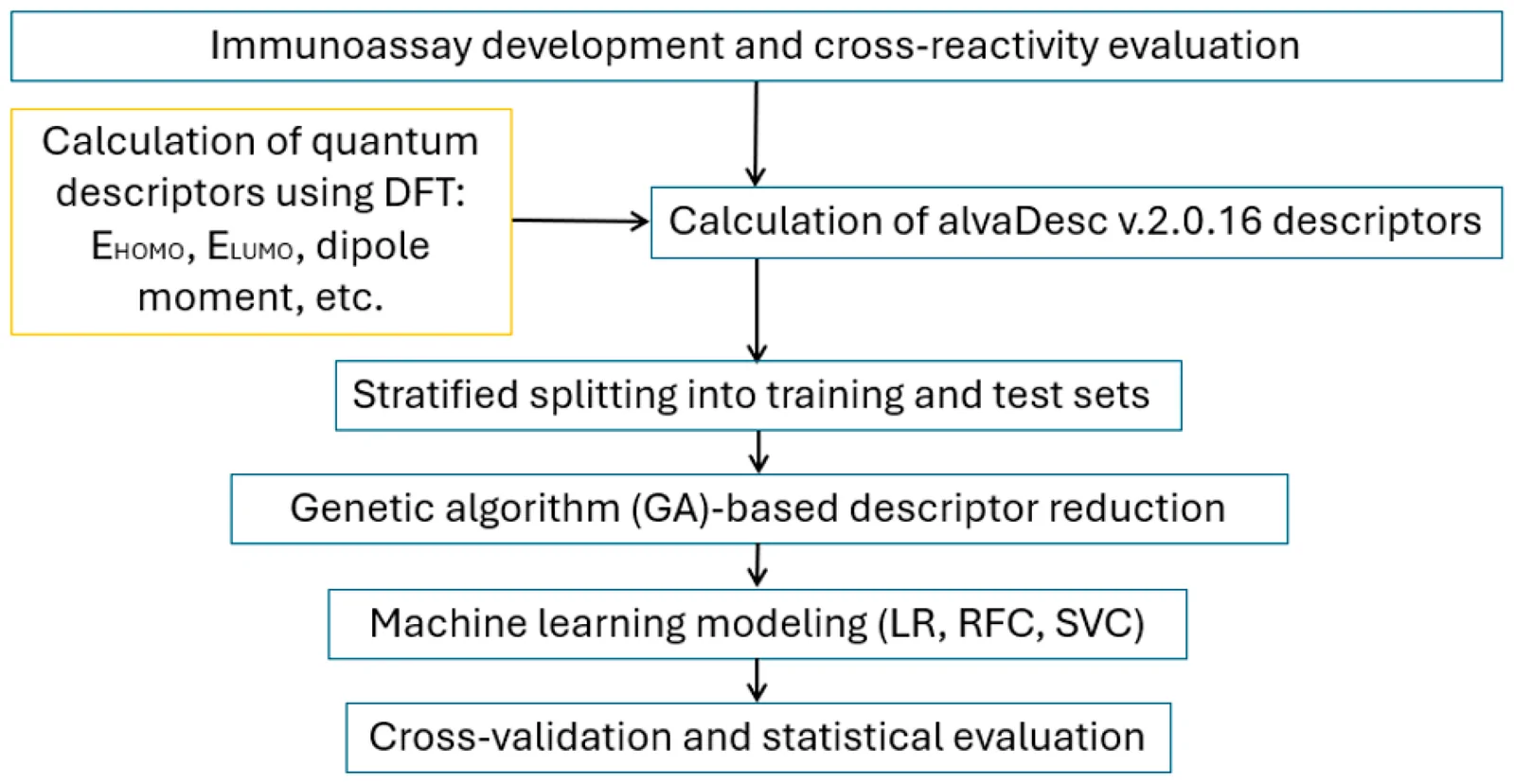
Workflow of cross-reactivity modeling using structure–activity relationship.

Using the OCHEM online platform [25], alvaDesc v.2.0.16 descriptors for FQ molecules were calculated: a total of >5000 molecular descriptors (Figure 2). Duplicated and highly correlated (R > 0.9) descriptors were removed. The dataset was subsequently divided into training and test sets using stratified sampling (test size = 20%) to preserve the distribution of the response variable. The descriptors of the training set were divided into blocks of 100 features, and 5 features were selected from each block using a genetic algorithm (GA) [26,27]. Quantum-chemical descriptors (HOMO orbital energy, LUMO orbital energy, dipole moment, electronegativity, polarizability, etc.—a total of 16 descriptors) were added to the analysis. GA was finally applied to the resulting feature, leaving six descriptors for a dataset of 26 molecules.

### 3.6. Classification

The class value was set to 1 or 0 using a cutoff of CR = 0.7%. The dataset was split into a test and a training set. A trial model was developed for the training and test set, and its parameters were determined (to test the model’s performance). Considering the stratified splitting, only one molecule of class “1” and five molecules of class “0” were included in the test set.

Next, CV was performed (to assess the quality of the model), and the averaged parameters have been calculated. For a reliable assessment on a small and unbalanced dataset (five molecules of class “1” and 21 molecules of class “0”), stratified repeated CV was applied. The data were divided into the training set and test set repeatedly in such a way that the class ratio was maintained. For each split, the model was trained, and then the probabilities of objects belonging to the positive class were predicted. To correct the class imbalance, the class_weight = ‘balanced’ parameter was used.

Internal CV (inner CV) was performed only on the training set, where the optimal classification threshold maximizing the F1 score was selected. This threshold was fixed and applied to the test set for the given split. For each split, statistical metrics were evaluated: Precision, Recall, F1 score, ROC-AUC, PR-AUC (average precision), and Log Loss. The resulting metric values were averaged across all splits and presented along with the standard deviation. This approach reduced the risk of overfitting and allowed us to obtain a robust assessment of the model quality, even with a limited number of positive examples.

### 3.7. Feature Importance

The contribution of descriptors was assessed after constructing final models trained on all available data. For the random forest, the Gini index (feature_importances_) was used, reflecting the contribution of each descriptor to reducing uncertainty when splitting the data. For linear models (logistic regression, linear SVC), normalized feature coefficients have been analyzed: the sign indicates the direction of influence on the target variable, and the magnitude indicates the strength of the contribution.

### 3.8. Calculation of Statistical Metrics

Precision (accuracy, positive predictive value) was obtained as follows:

(2) $$\mathrm{Precision}\;=\;\frac{TP}{TP+FP}$$

where TP is the true positive value, and FP is the false positive value.

Recall is a measure of sensitivity, or the proportion of correctly predicted positive objects among all actual positive ones:

(3) $$\mathrm{Recall}\;=\;\frac{TP}{TP+FN}$$

where FN is false negative.

F1-score is the harmonic mean of Precision and Recall:

(4) $$F1\;=\;2\;\times \;\frac{Precision\times Recall}{Precision+Recall}$$

ROC-AUC (area under the ROC curve) evaluates the quality of the model at different classification thresholds and shows the ability to distinguish between classes.

PR-AUC (area under the precision-recall curve) characterizes the model’s ability to identify the positive class when varying the classification threshold. Unlike ROC-AUC, it is more informative when the number of positive-class objects is small. Log Loss is used to evaluate probabilistic classification models. Log Loss penalizes the model for inaccurate probabilistic predictions.

## 4. Conclusions

Overall, five polyclonal antibodies were produced for the development of immunoassay systems using S-OFL–BSA, R-OFL–BSA, S-OFL-GABA–BSA, S-OFL–STI, and S, R-OFL–STI as immunogens. Their *IC50* values and CRs were evaluated. The results demonstrate that the immunoassays are highly sensitive to molecular structure and effectively distinguish between antibiotics, with most FQs exhibiting CR values below 0.1%. Consequently, structure–activity relationship classifiers were employed instead of quantitative regression models. Regarding machine learning procedures, CV is essential for an objective assessment of model performance. Among the tested methods, the support vector classifier provided the lowest Log Loss in four out of five immunogen systems, whereas the random forest classifier method performed best for the AS2 system, which utilized the S, R-OFL–STI immunogen. Overall, logistic regression offered the best balance between predictive accuracy and robustness to class imbalance. Descriptor analysis indicated that spatial and topological features, as well as the presence of aromatic hydroxyl groups, are key determinants of immunochemical activity and were consistently exploited across multiple models. Furthermore, 3D autocorrelation (R3m), 3D-MoRSE signals (Mor24u and Mor24v), and radial distribution functions (RDF105v, RDF035v, and RDF130m) proved valuable for differentiating stereoisomers and should be incorporated in future (Q)SAR and ML modeling efforts. These computational models collectively demonstrate that both structural parameters and spatial molecular interactions significantly influence CR in FQ immunoassays.

## Figures and Tables

**Table 1:** CRs of polyclonal antibodies toward FQs.

FQ	AS1 (Immunogen: S-OFL–STI)		AS2 (Immunogen: S, R-OFL–STI)	
	*IC50*, ng/mL	CR, %	*IC50*, ng/mL	CR, %
S-OFL	0.089 ± 0.024	100	0.336 ± 0.061	83.3
S, R-OFL^test^	0.034 ± 0.013	262	0.28 ± 0.03	100
GAR	0.28 ± 0.039	31.8	3.625 ± 0.84	7.7
MAR	1.278 ± 0.094	7.0	2.322 ± 0.433	12.1
RUF	0.034 ± 0.008	262	1.04 ± 0.136	26.9
CIN	11.084 ± 6.415	0.8	36.307 ± 9.676	0.77
PEF	44.975 ± 3.257	0.2	15.236 ± 4.495	1.8
ENR	24.741 ± 2.183	0.36	26.56 ± 9.805	1.1
GAT	>10,000	<0.01	>10,000	<0.01
ORB	>10,000	<0.01	>10,000	<0.01
LOM	>10,000	<0.01	>10,000	<0.01
SPAR^test^	>10,000	<0.01	>10,000	<0.01
ENO^test^	>10,000	<0.01	>10,000	<0.01
NAD	>10,000	<0.01	>10,000	<0.01
TOS	>10,000	<0.01	>10,000	<0.01
MOX^test^	>10,000	<0.01	>10,000	<0.01
DAN	>10,000	<0.01	>10,000	<0.01
CLI	>10,000	<0.01	>10,000	<0.01
CIP	>10,000	<0.01	>10,000	<0.01
DIF^test^	>10,000	<0.01	>10,000	<0.01
PAZ	>10,000	<0.01	>10,000	<0.01
SAR	>10,000	<0.01	>10,000	<0.01
FLU	>10,000	<0.01	>10,000	<0.01
PIP	>10,000	<0.01	>10,000	<0.01
NAL^test^	>10,000	<0.01	>10,000	<0.01
OXO	>10,000	<0.01	>10,000	<0.01

Values of *IC50* are presented as mean ± SD (n = 3). CR values were calculated using the mean *IC50* values. Compounds marked as “test” were included in the test set for structure–activity modeling.

**Table 2:** CRs of the second portion of polyclonal antibodies toward FQs.

FQ	AS3 (Immunogen: S-OFL-BSA)		AS4 (Immunogen: R-OFL-BSA)		AS5 (Immunogen: S-OFL-GABA–BSA)	
	*IC50*, ng/mL	CR, %	*IC50*, ng/mL	CR, %	*IC50*, ng/mL	CR, %
S-OFL	0.97 ± 0.08	100	0.04 ± 0.006	75	0.12 ± 0.02	100
R-OFL^test^	5.74 ± 0.53	16.9	0.03 ± 0.004	100	0.74 ± 0.12	16.2
GAR	13.5 ± 2.93	7.2	0.04 ± 0.003	75	1.3 ± 0.14	9.2
MAR	6.3 ± 0.54	15.4	0.03± 0.005	100	0.93 ± 0.22	12.9
RUF	4.76 ± 0.50	20.4	0.1 ± 0.009	30	0.35 ± 0.05	34.3
PEF	149.3 ± 57.5	0.65	16.1 ± 1.1	0.19	42.8 ± 7.4	0.28
ENR	78.0 ± 12.0	1.24	11.7 ± 1.3	0.34	82.4 ± 41.9	0.14
DIF	119.4 ± 72.8	0.81	120.3 ± 35.2	0.025	52.9 ± 15.0	0.23
LOM	>10,000	<0.01	189.5 ± 50.9	0.015	>10,000	<0.01
CIN	>10,000	<0.01	>10,000	<0.01	>10,000	<0.01
GAT	>10,000	<0.01	>10,000	<0.01	>10,000	<0.01
ORB^test^	>10,000	<0.01	>10,000	<0.01	>10,000	<0.01
SPAR^test^	>10,000	<0.01	>10,000	<0.01	>10,000	<0.01
ENO	>10,000	<0.01	>10,000	<0.01	>10,000	<0.01
NAD	>10,000	<0.01	>10,000	<0.01	>10,000	<0.01
TOS^test^	>10,000	<0.01	>10,000	<0.01	>10,000	<0.01
MOX	>10,000	<0.01	>10,000	<0.01	>10,000	<0.01
DAN	>10,000	<0.01	>10,000	<0.01	>10,000	<0.01
CLI	>10,000	<0.01	>10,000	<0.01	>10,000	<0.01
CIP^test^	>10,000	<0.01	>10,000	<0.01	>10,000	<0.01
PAZ	>10,000	<0.01	>10,000	<0.01	>10,000	<0.01
SAR	>10,000	<0.01	>10,000	<0.01	>10,000	<0.01
FLU	>10,000	<0.01	>10,000	<0.01	>10,000	<0.01
PIP	>10,000	<0.01	>10,000	<0.01	>10,000	<0.01
NAL^test^	>10,000	<0.01	>10,000	<0.01	>10,000	<0.01
OXO	>10,000	<0.01	>10,000	<0.01	>10,000	<0.01

Values of *IC50* are presented as mean ± SD (n = 3). CR values were calculated using the mean *IC50* values. Compounds marked as “test” were included in the test set for structure–activity modeling.

**Table 3:** Statistical metrics of the structure–activity relationship models for the AS1 system.

Model	Precision	Recall	F1-Score	ROC-AUC	PR-AUC	Log Loss
RFC	1.0	1.0	1.0	1.0	1.0	0.086
RFC + CV	0.627 ± 0.398	0.810 ± 0.392	0.679 ± 0.379	0.984 ± 0.065	0.968 ± 0.126	0.199 ± 0.106
LR	1.0	1.0	1.0	1.0	1.0	0.117
LR + CV	0.855 ± 0.234	1.000 ± 0.000	0.902 ± 0.160	1.000 ± 0.000	1.000 ± 0.000	0.168 ± 0.071
SVC	1.0	1.0	1.0	1.0	1.0	0.087
SVC + CV	0.745 ± 0.375	0.850 ± 0.357	0.778 ± 0.357	0.993 ± 0.040	0.985 ± 0.085	0.153 ± 0.095

**Table 4:** Descriptors and their contribution to structure–activity models of the AS1 system.

Descriptor	Feature Importance, %			Description
	RFC	LR	SVC	
JGI5	27.6	−17.5	−19.9	Mean topological charge index of order 5
RDF130m	22.5	22.2	16.0	Radial distribution function—130/weighted by mass
E_total_ (au)	13.7	−10.1	−14.9	Total energy in arbitrary units
CATS2D_02_AL	17.8	−17.9	−19.5	CATS2D acceptor-lipophilic at lag 02
nRNR2	9.3	15.3	15.7	Number of tertiary amines (aliphatic)
VE1_D/Dt	9.0	−17.0	−14.1	Coefficient sum of the last eigenvector from the distance/detour matrix

**Table 5:** Statistical metrics for the AS2 system.

Model	Precision	Recall	F1-Score	ROC-AUC	PR-AUC	Log Loss
RFC	1.0	1.0	1.0	1.0	1.0	0.122
RFC + CV	0.624 ± 0.411	0.770 ± 0.421	0.669 ± 0.400	0.974 ± 0.080	0.948 ± 0.156	0.214 ± 0.135
LR	0.5	1.0	0.7	1.0	1.0	0.271
LR + CV	0.666 ± 0.414	0.780 ± 0.414	0.701 ± 0.402	0.950 ± 0.124	0.907 ± 0.207	0.282 ± 0.170
SVC	1.0	1.0	1.0	1.0	1.0	0.173
SVC + CV	0.610 ± 0.409	0.780 ± 0.414	0.659 ± 0.396	0.921 ± 0.168	0.877 ± 0.248	0.287 ± 0.189

**Table 6:** Molecular descriptors and their contribution to computational models for the AS2 system.

Descriptor	Feature Importance, %			Description
	RFC	LR	SVC	
VE1_D/Dt	41.8	−26.1	−19.4	coefficient sum of the last eigenvector from distance/detour matrix
CATS3D_11_AA	18.6	27.1	34.9	H-bond acceptor—acceptor pairs at 11 Å
RDF105v	12.9	−5.8	0.01	Radial Distribution Function at 10.5 Å weighted by van der Waals volume
PW4	11.0	15.5	21.2	Path/walk connectivity index of order 4
MDEN-23	10.8	−5.3	1.2	Molecular distance edge descriptor No. 23 (edge adjacency/topological distance index)
qpmax	4.9	20.3	23.3	Maximum atomic positive partial charge

**Table 7:** Statistical metrics of the structure–activity models for the AS3 system.

Model	Precision	Recall	F1-Score	ROC-AUC	PR-AUC	Log Loss
RFC	1.0	1.0	1.0	1.0	1.0	0.143
RFC + CV	0.661 ± 0.380	0.840 ± 0.367	0.716 ± 0.358	0.985 ± 0.062	0.968 ± 0.126	0.238 ± 0.064
LR	1.0	1.0	1.0	1.0	1.0	0.084
LR + CV	0.910 ± 0.213	0.990 ± 0.099	0.935 ± 0.163	1.000 ± 0.000	1.000 ± 0.000	0.179 ± 0.070
SVC	1.0	1.0	1.0	1.0	1.0	0.028
SVC + CV	0.878 ± 0.243	0.990 ± 0.099	0.912 ± 0.185	1.000 ± 0.000	1.000 ± 0.000	0.122 ± 0.073

**Table 8:** Molecular descriptors and their contributions to structure–activity models of the AS3 system.

Descriptor	Feature Importance, %			Description
	RFC	LR	SVC	
Eig05_EA(dm)	38.6	−17.5	35.0	5th eigenvalue of the edge adjacency matrix from distance/detour matrix
CATS3D_11_AA	15.7	22.2	24.1	H-bond acceptor—acceptor pairs at 11 Å
R3m	12.6	−2.1	−13.9	3D autocorrelation at lag 3 Å weighted by atomic mass
Mor24v	11.8	9.0	1.5	3D-MoRSE signal 24 weighted by van der Waals volume
E_LUMO_ (eV)	11.1	−7.5	−15.8	LUMO energy in eV
SsssCH	10.2	−18.6	−9.8	Sum of E-state indices for >CH– groups (tertiary carbon)

**Table 9:** Statistical metrics of the AS4 system.

Model	Precision	Recall	F1-Score	ROC-AUC	PR-AUC	Log Loss
RFC	1.0	1.0	1.0	1.0	1.0	0.058
RFC + CV	0.827 ± 0.287	0.960 ± 0.196	0.868 ± 0.240	1.000 ± 0.000	1.000 ± 0.000	0.145 ± 0.074
LR	1.0	1.0	1.0	1.0	1.0	0.113
LR + CV	0.932 ± 0.178	1.000 ± 0.000	0.953 ± 0.123	1.000 ± 0.000	1.000 ± 0.000	0.152 ± 0.067
SVC	1.0	1.0	1.0	1.0	1.0	0.056
SVC + CV	0.915 ± 0.188	1.000 ± 0.000	0.943 ± 0.125	1.000 ± 0.000	1.000 ± 0.000	0.104 ± 0.049

**Table 10:** Descriptors and their contributions to the computational models of the AS4 system.

Descriptor	Feature Importance, %			Description
	RFC	LR	SVC	
Eig05_EA(dm)	29.9	32.7	35.6	5th eigenvalue of the edge adjacency matrix from distance/detour matrix
VE2_D/Dt	36.9	−14.0	−16.3	Coefficient sum of the 2nd eigenvector from distance/detour matrix
SsssCH	8.4	−19.2	−21.2	Sum of E-state indices for –CH– groups (tertiary carbon)
N-068	9.4	17.1	16.0	number of hydroxyl groups attached to aromatic rings (Ar–OH)
CATS3D_03_AN	4.5	11.7	7.9	3D CATS descriptor: frequency of Acceptor–Negative pharmacophore pairs at distance 3 Å
Mor24u	11.0	5.4	−3.0	3D-MoRSE signal 24 (unweighted)

**Table 11:** Statistical metrics for the S-OFL-GABA–BSA immunogen.

Model	Precision	Recall	F1-Score	ROC-AUC	PR-AUC	Log Loss
RFC	1.0	1.0	1.0	1.0	1.0	0.104
RFC + CV	0.545 ± 0.425	0.700 ± 0.458	0.592 ± 0.421	0.967 ± 0.101	0.937 ± 0.182	0.251 ± 0.171
LR	1.0	1.0	1.0	1.0	1.0	0.094
LR + CV	0.888 ± 0.288	0.930 ± 0.255	0.900 ± 0.271	1.000 ± 0.000	1.000 ± 0.000	0.166 ± 0.073
SVC	1.0	1.0	1.0	1.0	1.0	0.076
SVC + CV	0.725 ± 0.392	0.830 ± 0.376	0.757 ± 0.375	0.998 ± 0.025	0.995 ± 0.050	0.185 ± 0.106

**Table 12:** Descriptors and their contributions to the computational models of the S-OFL-GABA–BSA immunogen.

Descriptor	Feature Importance, %			Description
	RFC	LR	SVC	
Eig05_EA(dm)	38.8	40.8	39.4	5th eigenvalue of the edge adjacency matrix from distance/detour matrix
SsssCH	13.5	−27.0	−23.3	Sum of E-state indices for –CH– groups (tertiary carbon)
N-068	9.4	19.0	30.4	Number of hydroxyl groups attached to aromatic rings (Ar–OH)
CATS3D_11_AA	14.7	8.2	−3.7	H-bond acceptor—acceptor pairs at 11 Å
RDF035v	15.1	−3.3	−0.9	Radial Distribution Function at 3.5 Å weighted by van der Waals volume
C-006	8.5	1.6	−2.3	E-state index for –CH_2_– groups (aliphatic carbon)

## Data Availability

The original contributions of this study are included in this manuscript, and further inquiries can be directed to the corresponding author.

## Supplementary Materials

The following supporting information can be downloaded at https://www.mdpi.com/article/10.3390/ijms27188140/s1.

## Abbreviations

The following abbreviations are used in this manuscript:

CR	Cross-reactivity
CV	Cross-validation
FQ	Fluoroquinolone
LR	Logistic regression
ML	Machine learning
OFL	Ofloxacin
RFC	Random forest classifier
SVC	Support vector classifier
