# Supplementary material for: Structure–Activity Relationship Analysis of Immunoassay Systems for (Fluoro)quinolones Detection
Source: Int J Mol Sci. 2026 Sep 12;27(18):8140. doi: 10.3390/ijms27188140 (PMC13607221; doi:10.3390/ijms27188140)
Supplement: Supplementary file 1 [file ijms-27-08140-s001.zip › ijms-4513104-supplementary.pdf]

# Structure-Activity Relationship Analysis of Immunoassay Systems for (Fluoro)quinolones Detection

**Platon P. Chebotaev [1], Andrey A. Buglak [1,2,*], Nadezhda A. Byzova [3], Anatoly V. Zherdev [3] and Olga D. Hendrickson [3]**

[1] Department of Molecular Biophysics and Polymer Physics, St. Petersburg State University, 7/9 Universitetskaya Embankment, 199034 St. Petersburg, Russia; st068500@student.spbu.ru
[2] Department of Quantum Electronics and Radiospectroscopy, Institute of Physics, Kazan Federal University, Kremlevskaya str. 18, 420008 Kazan, Russia
[3] A.N. Bach Institute of Biochemistry, Research Center of Biotechnology, Russian Academy of Sciences, Leninsky prospect 33, 119071 Moscow, Russia; nbyzova@inbi.ras.ru (N.A.B.); zherdev@inbi.ras.ru (A.V.Z.); odhendrick@gmail.com (O.D.H.)

* Correspondence: andreybuglak@gmail.com

# Electronic supplement

**Table S1.** Experimental conditions during the synthesis of immunogens and coating antigens.

| Reagent | S-OFL–BSA | R-OFL–BSA | S-OFL-GABA–BSA | S-OFL-GABA–OVA | S-OFL–STI | S,R-OFL–STI |
|---|---|---|---|---|---|---|
| Hapten, mg | 2.5 | 2.5 | 3 | 3 | 2.5 | 2.5 |
| NHS, mg | 1.7 | 1.7 | 2.3 | 2.3 | 1.7 | 1.7 |
| EDC, mg | 2.9 | 2.9 | no | no | 2.9 | 2.9 |
| DCC, mg | no | no | 2.6 | 2.6 | no | no |
| GABA, mg | no | no | 2.2 | 2.2 | no | no |
| BSA, mg | 5.5 | 5.5 | 5.5 | no | no | no |
| STI, mg | no | no | no | no | 7 | 7 |
| OVA, mg | no | no | no | 5 | no | no |

**Table S2.** Molecular descriptor values for the AS1 system.

| Compound | nRNR2 | JGI5 | CATS2D_02_AL | RDF130m | $E_{Total}$ (au) | VE1_D/Dt |
|---|---|---|---|---|---|---|
| S-ofloxacin (S-OFL) 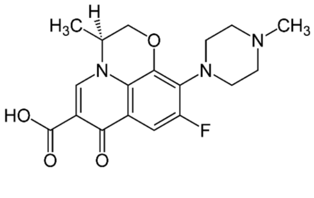 | 1 | 0.03216 | 7 | 1.245 | -1262.46 | 3.862 |
| S, R-ofloxacin (S, R-OFL) test 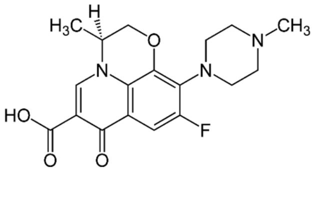 | 1 | 0.03216 | 7 | 1.344 | -1262.46 | 3.862 |
| Garenofloxacin (GAR) 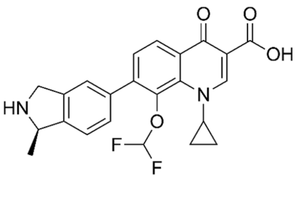 | 0 | 0.03125 | 9 | 3.738 | -1497.99 | 4.828 |

| | | | | | | |
|---|---|---|---|---|---|---|
| Marbofloxacin (MAR) 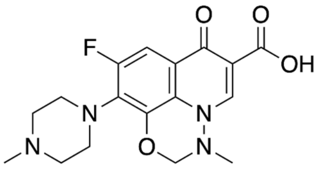 | 1 | 0.03216 | 6 | 0.00591 | -1278.45 | 3.862 |
| Rufloxacin (RUF) 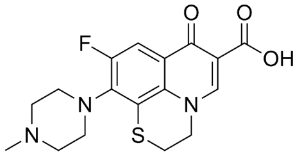 | 1 | 0.02778 | 6 | 0.7389 | -1546.13 | 3.828 |
| Cinoxacin (CIN) 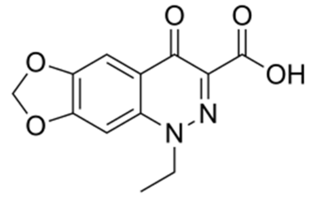 | 0 | 0.03499 | 6 | 0 | -948.522 | 3.774 |
| Pefloxacin (PEF) 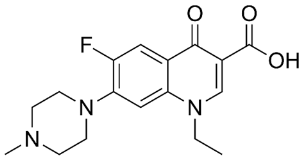 | 1 | 0.038 | 9 | 0.01001 | -1149.17 | 4.439 |
| Enrofloxacin (ENR) 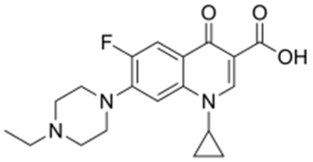 | 1 | 0.03455 | 11 | 1.136 | -1226.53 | 4.603 |
| Gatifloxacin (GAT) 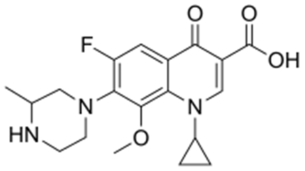 | 0 | 0.03195 | 8 | 0.00596 | -1301.7 | 4.675 |
| Orbifloxacin (ORB) | 0 | 0.03604 | 8 | 0.01417 | -1424.92 | 4.731 |

| | | | | | | |
|---|---|---|---|---|---|---|
| 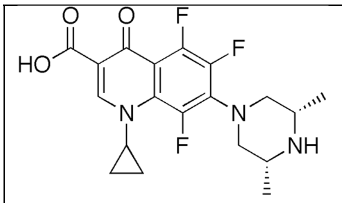 | | | | | | |
| Lomefloxacin (LOM) 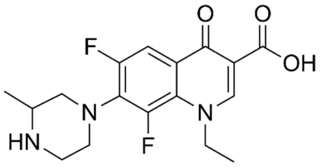 | 0 | 0.03604 | 7 | 0.00128 | -1248.37 | 4.514 |
| Sparfloxacin (SPAR) [test] 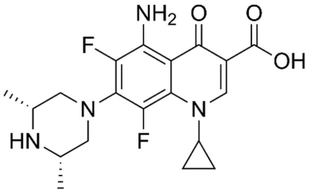 | 0 | 0.03604 | 8 | 0.01989 | -1381.07 | 4.731 |
| Enoxacin (ENO) [test] 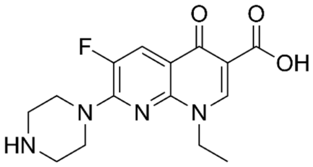 | 0 | 0.03591 | 8 | 0 | -1125.93 | 4.363 |
| Nadifloxacin (NAD) 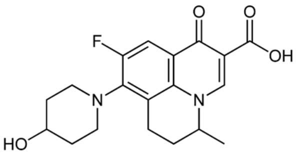 | 0 | 0.03216 | 14 | 0.00724 | -1246.45 | 3.862 |
| Tosufloxacin (TOS) 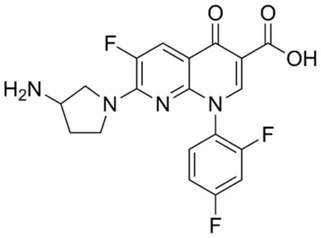 | 0 | 0.03446 | 9 | 0.00015 | -1476.7 | 4.679 |
| Moxifloxacin (MOX) [test] | 0 | 0.03386 | 9 | 0.27 | -1379.09 | 4.905 |

| | | | | | | |
|---|---|---|---|---|---|---|
| 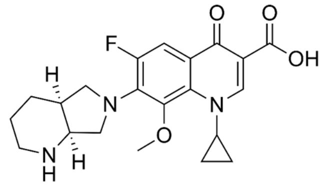 | | | | | | |
| Danofloxacin (DAN) 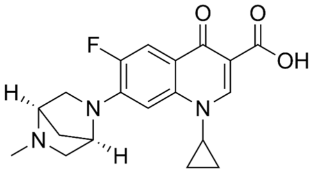 | 1 | 0.03327 | 12 | 0 | -1225.32 | 4.698 |
| Clinafloxacin (CLI) 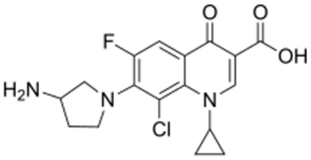 | 0 | 0.03643 | 9 | 0.08242 | -1607.49 | 4.557 |
| Ciprofloxacin (CIP) 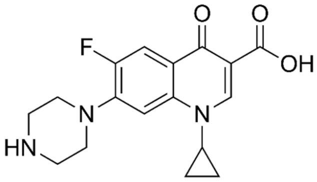 | 0 | 0.03187 | 10 | 0 | -1147.94 | 4.457 |
| Difloxacin (DIF) [test] 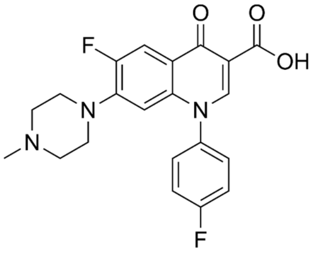 | 1 | 0.03401 | 10 | 0.03644 | -1400.75 | 4.773 |
| Pazufloxacin (PAZ) 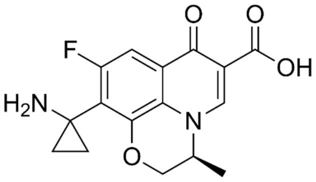 | 0 | 0.03737 | 8 | 0 | -1128.53 | 4.161 |

| | | | | | | |
|---|---|---|---|---|---|---|
| Sarafloxacin (SAR) 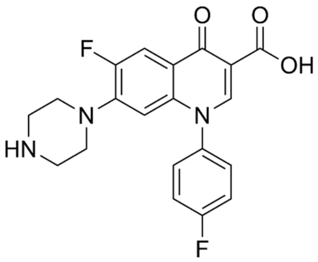 | 0 | 0.03241 | 10 | 0.00006 | -1361.45 | 4.659 |
| Flumequine (FLU) 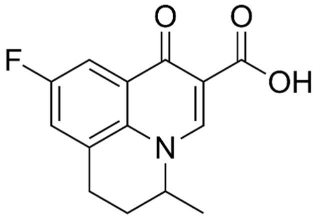 | 0 | 0.03739 | 9 | 0 | -920.663 | 3.626 |
| Pipemidic acid (PIP) 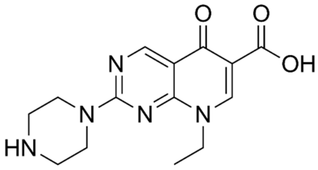 | 0 | 0.03634 | 9 | 0 | -1042.77 | 4.17 |
| Nalidixic acid (NAL) [test] 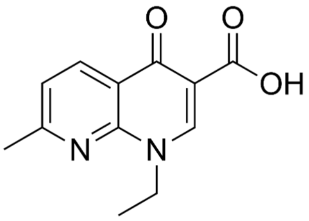 | 0 | 0.04201 | 10 | 0 | -799.399 | 3.457 |
| Oxolinic acid (OXO) 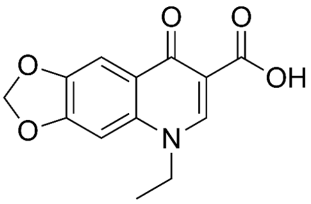 | 0 | 0.03499 | 10 | 0 | -932.523 | 3.774 |

[test] – test set

**Table S3.** Molecular descriptor values of the AS2 system.

| Compound | CATS3D_11_AA | qpmax | PW4 | VE2_D/Dt | RDF105v | MDEN-23 |
|---|---|---|---|---|---|---|
| S-ofloxacin (S-OFL) | 2 | 0.342 | 0.2044 | 0.1485 | 0.6824 | 0 |
| S, R-ofloxacin (S, R-OFL) [test] | 2 | 0.342 | 0.2044 | 0.1485 | 1.1417 | 0 |
| Garenofloxacin (GAR) | 0 | 0.3893 | 0.1958 | 0.1557 | 2.122 | 0.125 |
| Marbofloxacin (MAR) | 2 | 0.342 | 0.2044 | 0.1485 | 3.817 | 0 |
| Rufloxacin (RUF) | 2 | 0.342 | 0.2067 | 0.1531 | 1.323 | 0 |
| Cinoxacin (CIN) | 0 | 0.3609 | 0.2046 | 0.1986 | 0.0497 | 1 |
| Pefloxacin (PEF) | 2 | 0.342 | 0.193 | 0.1849 | 2.212 | 0 |
| Enrofloxacin (ENR) | 2 | 0.342 | 0.1854 | 0.177 | 1.536 | 0 |
| Gatifloxacin (GAT) | 0 | 0.342 | 0.2056 | 0.1731 | 1.405 | 0.4364 |
| Orbifloxacin (ORB) | 0 | 0.342 | 0.2046 | 0.169 | 2.888 | 0.4364 |

| | | | | | | |
|---|---|---|---|---|---|---|
| Lomefloxacin (LOM) | 0 | 0.342 | 0.2094 | 0.1805 | 1.4 | 0.4364 |
| Sparfloxacin (SPAR) test | 0 | 0.342 | 0.2046 | 0.169 | 3.206 | 0.4364 |
| Enoxacin (ENO) test | 0 | 0.342 | 0.2006 | 0.1897 | 0.7089 | 1.321 |
| Nadifloxacin (NAD) | 0 | 0.342 | 0.2044 | 0.1485 | 2.123 | 0 |
| Tosufloxacin (TOS) | 0 | 0.342 | 0.1954 | 0.1614 | 0.6378 | 1 |
| Moxifloxacin (MOX) test | 0 | 0.342 | 0.2107 | 0.1691 | 2.974 | 0.4364 |
| Danofloxacin (DAN) | 0 | 0.342 | 0.198 | 0.1807 | 0 | 0 |
| Clinafloxacin (CLI) | 0 | 0.342 | 0.2008 | 0.1823 | 0.5121 | 0 |
| Ciprofloxacin (CIP) | 0 | 0.342 | 0.1907 | 0.1857 | 1.074 | 0.4364 |
| Difloxacin (DIF) test | 2 | 0.342 | 0.1854 | 0.1646 | 4.048 | 0 |
| Pazufloxacin (PAZ) | 0 | 0.342 | 0.2018 | 0.1809 | 0.4983 | 0 |
| Sarafloxacin (SAR) | 0 | 0.342 | 0.1914 | 0.1664 | 1.324 | 0.4364 |

| | | | | | | |
|---|---|---|---|---|---|---|
| Flumequine (FLU) | 0 | 0.342 | 0.2066 | 0.1908 | 0 | 0 |
| Pipemidic acid (PIP) | 0 | 0.342 | 0.1971 | 0.1896 | 1.706 | 2.027 |
| Nalidixic acid (NAL) [test] | 0 | 0.342 | 0.1973 | 0.2034 | 0.00798 | 0.5 |
| Oxolinic acid (OXO) | 0 | 0.342 | 0.2046 | 0.1986 | 0 | 0 |

[test] – test set

**Table S4.** Molecular descriptor values of the AS3 system.

| Compound | Eig05_EA (dm) | CATS3D_11_AA | R3m | Mor24v | $E_{LUMO}$ (eV) | SsssCH |
|---|---|---|---|---|---|---|
| S-ofloxacin (S-OFL) 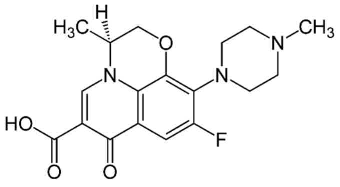 | 0.8 | 2 | 0.6439 | 0.2152 | -0.6470 | -0.1579 |
| R-ofloxacin (R-OFL) [test] 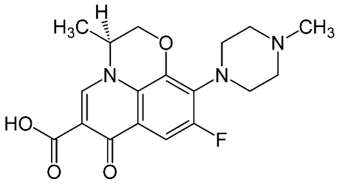 | 0.8 | 2 | 0.6644 | 0.0447 | -0.7016 | -0.1579 |
| Garenofloxacin (GAR) 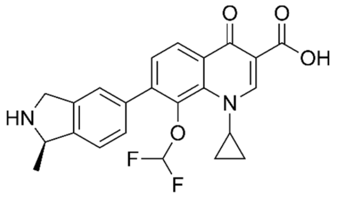 | 0.3586 | 0 | 0.6117 | 0.4340 | -0.7859 | -2.9340 |
| Marbofloxacin (MAR) | 0.4 | 2 | 0.6166 | 0.1102 | -0.6936 | 0.0000 |

| | | | | | | |
|---|---|---|---|---|---|---|
| 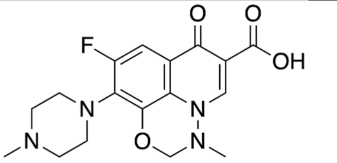 | | | | | | |
| Rufloxacin (RUF) 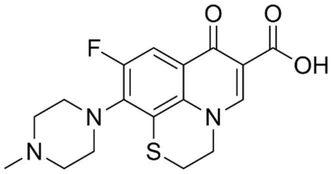 | 0.8 | 2 | 0.7579 | 0.1058 | -0.7586 | 0.0000 |
| Pefloxacin (PEF) 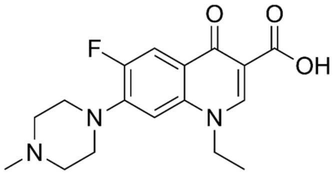 | 0 | 2 | 0.6235 | -0.0271 | -0.5818 | 0.0000 |
| Enrofloxacin (ENR) 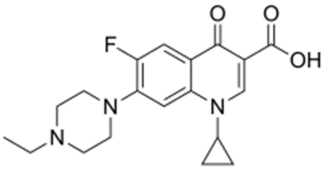 | 0 | 2 | 0.5623 | 0.2382 | -0.5260 | 0.1865 |
| Difloxacin (DIF) 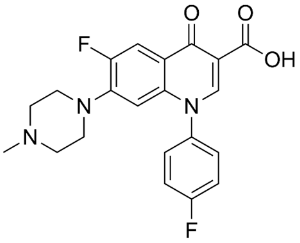 | 0 | 2 | 0.7195 | 0.0929 | -0.6547 | 0.0000 |
| Lomefloxacin (LOM) 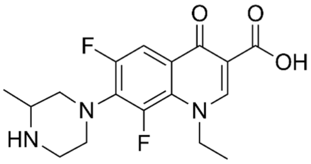 | 0 | 0 | 0.6779 | 0.1631 | -0.7513 | 0.0750 |
| Cinoxacin (CIN) 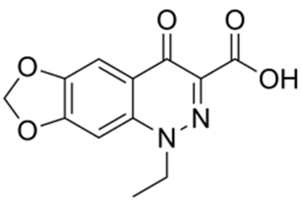 | 0 | 0 | 0.5745 | 0.0334 | -0.8991 | 0.0000 |
| Gatifloxacin (GAT) | 0.4 | 0 | 0.647 | 0.1271 | -0.6514 | 0.2907 |

| | | | | | | |
|---|---|---|---|---|---|---|
| 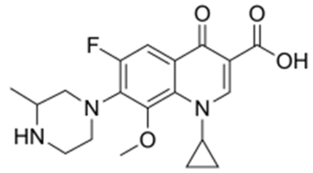 | | | | | | |
| Orbifloxacin (ORB) [test] 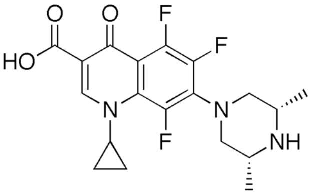 | 0 | 0 | 0.7704 | 0.0064 | -0.6569 | -0.3778 |
| Sparfloxacin (SPAR) [test] 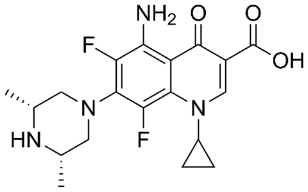 | 0 | 0 | 0.5994 | 0.2063 | -0.3960 | -0.1035 |
| Enoxacin (ENO) 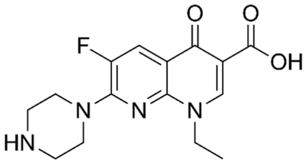 | 0 | 0 | 0.5615 | -0.0147 | -0.8729 | 0.0000 |
| Nadifloxacin (NAD) 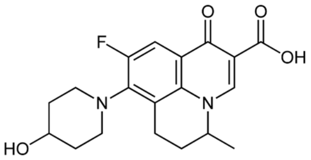 | 0 | 0 | 0.6671 | -0.0102 | -0.6339 | -0.3263 |
| Tosufloxacin (TOS) [test] 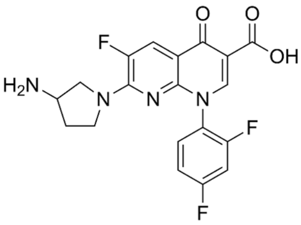 | 0 | 0 | 0.7521 | -0.0001 | -0.8246 | -0.1685 |
| Moxifloxacin (MOX) 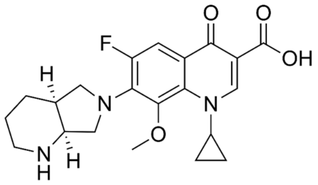 | 0.4 | 0 | 0.542 | -0.2686 | -0.5825 | 0.8874 |
| Danofloxacin (DAN) | 0 | 0 | 0 | 0.0000 | -0.4423 | 0.9186 |

| | | | | | | |
|---|---|---|---|---|---|---|
| 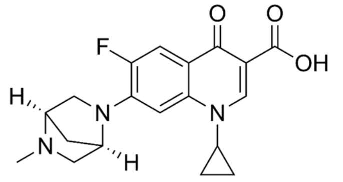 | | | | | | |
| Clinafloxacin (CLI) 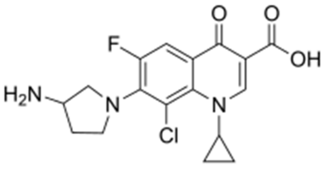 | 0 | 0 | 0.624 | 0.0748 | -0.8986 | 0.0315 |
| Ciprofloxacin (CIP) [test] 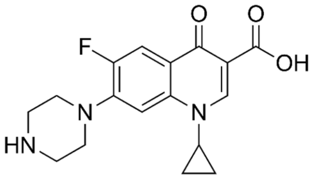 | 0 | 0 | 0.5467 | 0.1008 | -0.5234 | 0.1875 |
| Pazufloxacin (PAZ) 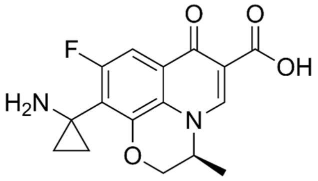 | 0 | 0 | 0.6197 | 0.2363 | -0.7214 | -0.1714 |
| Sarafloxacin (SAR) 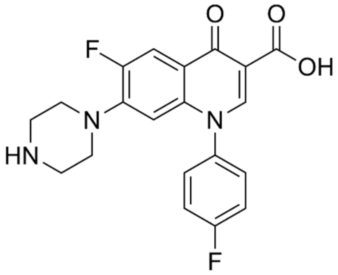 | 0 | 0 | 0.6748 | 0.2430 | -0.6449 | 0.0000 |
| Flumequine (FLU) 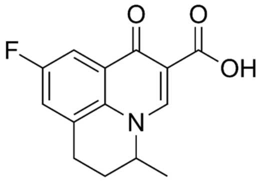 | 0 | 0 | 0.5982 | 0.0472 | -0.6770 | 0.0965 |
| Pipemidic acid (PIP) 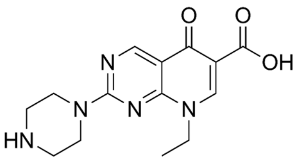 | 0 | 0 | 0.5772 | 0.0530 | -0.9288 | 0.0000 |
| Nalidixic acid (NAL) [test] | 0 | 0 | 0.5098 | -0.0030 | -0.8087 | 0.0000 |

| | | | | | | |
|---|---|---|---|---|---|---|
| 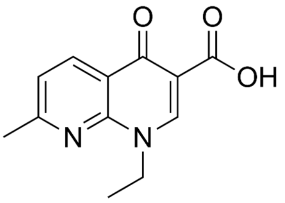 | | | | | | |
| Oxolinic acid (OXO) 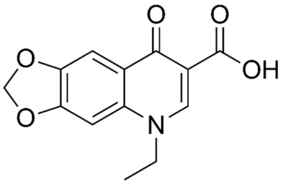 | 0 | 0 | 0.5656 | 0.1096 | -0.5066 | 0.0000 |

[test] – test set

**Table S5.** Molecular descriptor values of the AS4 system.

| Compound | Eig05_EA(dm) | VE2_D/Dt | SsssCH | N-068 | CATS3D_03_AN | Mor24u |
|---|---|---|---|---|---|---|
| S-ofloxacin (S-OFL) | 0.8 | 0.1485 | -0.1579 | 1 | 1 | 0.1724 |
| R-ofloxacin (R-OFL) [test] | 0.8 | 0.1485 | -0.1579 | 1 | 2 | 0.0822 |
| Garenofloxacin (GAR) | 0.3586 | 0.1557 | -2.934 | 0 | 1 | 0.3441 |
| Marbofloxacin (MAR) | 0.4 | 0.1485 | 0 | 2 | 2 | 0.1261 |
| Rufloxacin (RUF) | 0.8 | 0.1531 | 0 | 1 | 1 | -0.1930 |
| Pefloxacin (PEF) | 0 | 0.1849 | 0 | 1 | 1 | -0.2436 |
| Enrofloxacin (ENR) | 0 | 0.177 | 0.1865 | 1 | 1 | 0.3147 |
| Difloxacin (DIF) | 0 | 0.1646 | 0 | 1 | 1 | -0.1464 |
| Lomefloxacin (LOM) | 0 | 0.1805 | 0.075 | 0 | 1 | -0.0099 |
| Cinoxacin (CIN) | 0 | 0.1986 | 0 | 0 | 1 | -0.0828 |
| Gatifloxacin (GAT) | 0.4 | 0.1731 | 0.2907 | 0 | 1 | 0.2235 |
| Orbifloxacin (ORB) [test] | 0 | 0.169 | -0.3778 | 0 | 1 | -0.1126 |

| | | | | | | |
|---|---|---|---|---|---|---|
| Sparfloxacin (SPAR) [test] | 0 | 0.169 | -0.1035 | 0 | 1 | 0.1647 |
| Enoxacin (ENO) | 0 | 0.1897 | 0 | 0 | 1 | -0.1963 |
| Nadifloxacin (NAD) | 0 | 0.1485 | -0.3263 | 0 | 1 | -0.3120 |
| Tosufloxacin (TOS) [test] | 0 | 0.1614 | -0.1685 | 0 | 1 | -0.1817 |
| Moxifloxacin (MOX) | 0.4 | 0.1691 | 0.8874 | 0 | 1 | -0.6387 |
| Danofloxacin (DAN) | 0 | 0.1807 | 0.9186 | 1 | 0 | 0.0000 |
| Clinafloxacin (CLI) | 0 | 0.1823 | 0.0315 | 0 | 1 | -0.0993 |
| Ciprofloxacin (CIP) [test] | 0 | 0.1857 | 0.1875 | 0 | 1 | 0.0657 |
| Pazufloxacin (PAZ) | 0 | 0.1809 | -0.1714 | 0 | 1 | 0.0870 |
| Sarafloxacin (SAR) | 0 | 0.1664 | 0 | 0 | 1 | 0.1924 |
| Flumequine (FLU) | 0 | 0.1908 | 0.0965 | 0 | 1 | -0.0919 |
| Pipemidic acid (PIP) | 0 | 0.1896 | 0 | 0 | 1 | -0.2021 |
| Nalidixic acid (NAL) [test] | 0 | 0.2034 | 0 | 0 | 1 | -0.0482 |
| Oxolinic acid (OXO) | 0 | 0.1986 | 0 | 0 | 1 | -0.0125 |

[test] – test set

**Table S6.** Molecular descriptor values of the AS5 system.

| Compound | Eig05_EA(dm) | SsssCH | N-068 | CATS3D_11_AA | RDF035v | C-006 |
|---|---|---|---|---|---|---|
| S-ofloxacin (S-OFL) | 0.8 | 0.1485 | 1 | 2 | 4.016 | 5 |

| | | | | | | |
|---|---|---|---|---|---|---|
| R-ofloxacin (R-OFL) test | 0.8 | 0.1485 | 1 | 2 | 6.110 | 5 |
| Garenofloxacin (GAR) | 0.3586 | 0.1557 | 0 | 0 | 12.120 | 1 |
| Marbofloxacin (MAR) | 0.4 | 0.1485 | 2 | 2 | 4.232 | 4 |
| Rufloxacin (RUF) | 0.8 | 0.1531 | 1 | 2 | 4.141 | 6 |
| Pefloxacin (PEF) | 0 | 0.1849 | 1 | 2 | 4.098 | 5 |
| Enrofloxacin (ENR) | 0 | 0.177 | 1 | 2 | 8.653 | 5 |
| Difloxacin (DIF) | 0 | 0.1646 | 1 | 2 | 5.131 | 4 |
| Lomefloxacin (LOM) | 0 | 0.1805 | 0 | 0 | 7.171 | 4 |
| Cinoxacin (CIN) | 0 | 0.1986 | 0 | 0 | 5.862 | 1 |
| Gatifloxacin (GAT) | 0.4 | 0.1731 | 0 | 0 | 10.100 | 3 |
| Orbifloxacin (ORB) test | 0 | 0.169 | 0 | 0 | 7.951 | 2 |
| Sparfloxacin (SPAR) test | 0 | 0.169 | 0 | 0 | 7.807 | 2 |
| Enoxacin (ENO) | 0 | 0.1897 | 0 | 0 | 4.992 | 5 |
| Nadifloxacin (NAD) | 0 | 0.1485 | 0 | 0 | 9.712 | 2 |
| Tosufloxacin (TOS) test | 0 | 0.1614 | 0 | 0 | 4.514 | 2 |
| Moxifloxacin (MOX) | 0.4 | 0.1691 | 0 | 0 | 8.228 | 3 |
| Danofloxacin (DAN) | 0 | 0.1807 | 1 | 0 | 0.000 | 2 |
| Clinafloxacin (CLI) | 0 | 0.1823 | 0 | 0 | 6.363 | 2 |
| Ciprofloxacin (CIP) test | 0 | 0.1857 | 0 | 0 | 7.470 | 4 |

| | | | | | | |
|---|---|---|---|---|---|---|
| Pazufloxacin (PAZ) | 0 | 0.1809 | 0 | 0 | 5.481 | 1 |
| Sarafloxacin (SAR) | 0 | 0.1664 | 0 | 0 | 6.136 | 4 |
| Flumequine (FLU) | 0 | 0.1908 | 0 | 0 | 3.775 | 0 |
| Pipemidic acid (PIP) | 0 | 0.1896 | 0 | 0 | 7.264 | 5 |
| Nalidixic acid (NAL) [test] | 0 | 0.2034 | 0 | 0 | 2.633 | 1 |
| Oxolinic acid (OXO) | 0 | 0.1986 | 0 | 0 | 5.545 | 1 |

[test] – test set

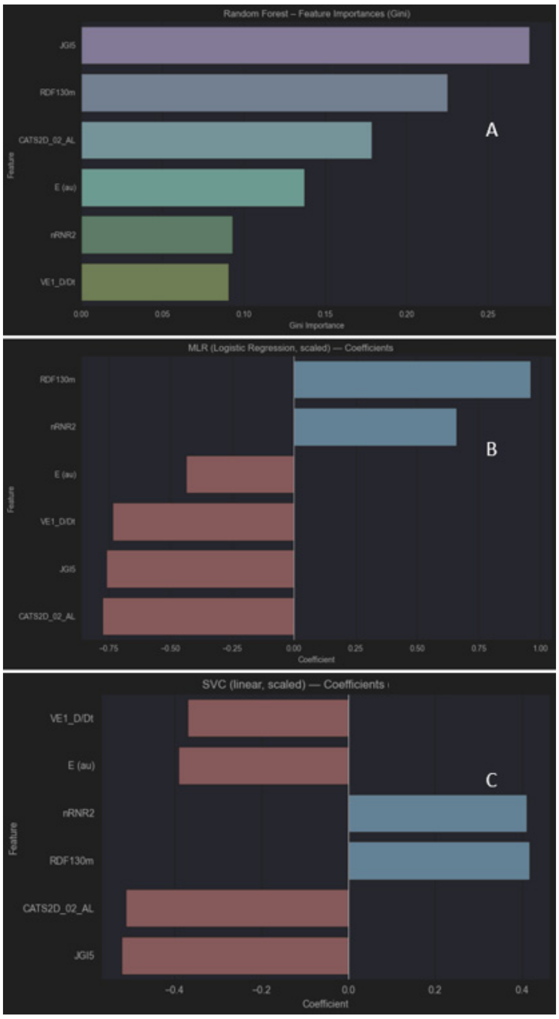


**Figure S1.** Feature importance of the AS1 system: RFC model (A), LR model (B), and SVC model (C).

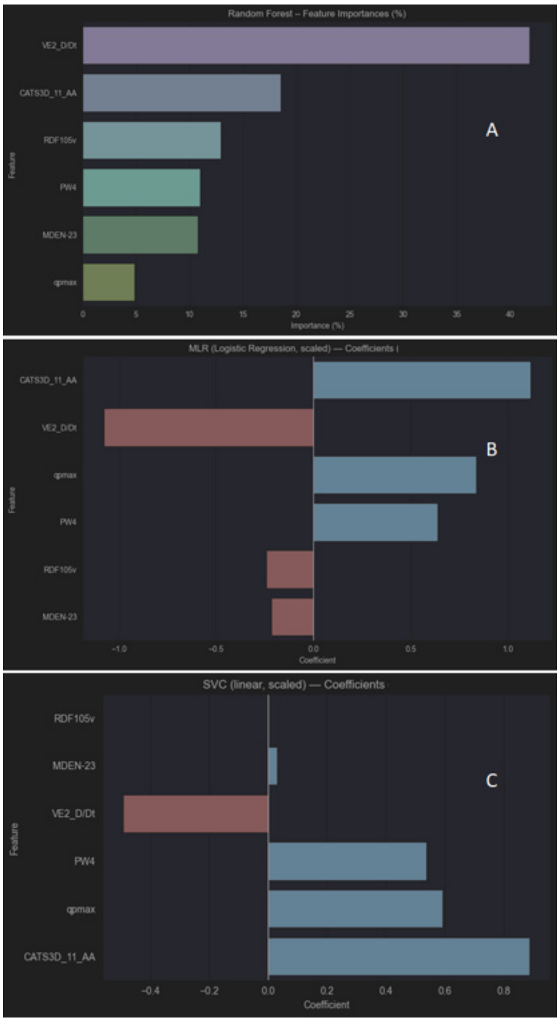


**Figure S2.** Feature importance for the RFC model (A), logistic regression (B), and SVC model (C) of the AS2 system.

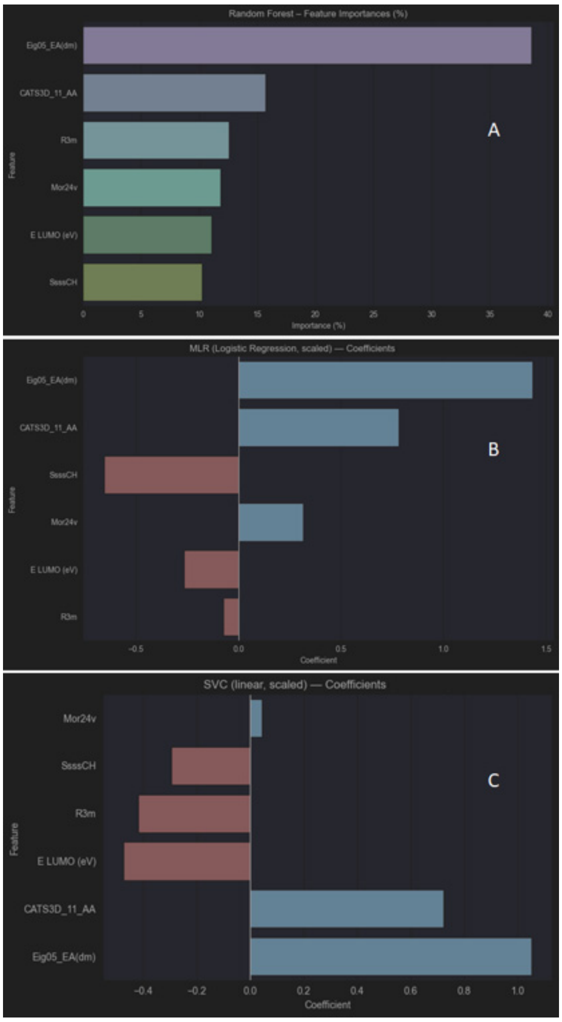


**Figure S3.** Contribution of descriptors to the random forest model (A), logistic regression (B), and support vector classifier (C) model of the AS3 system.

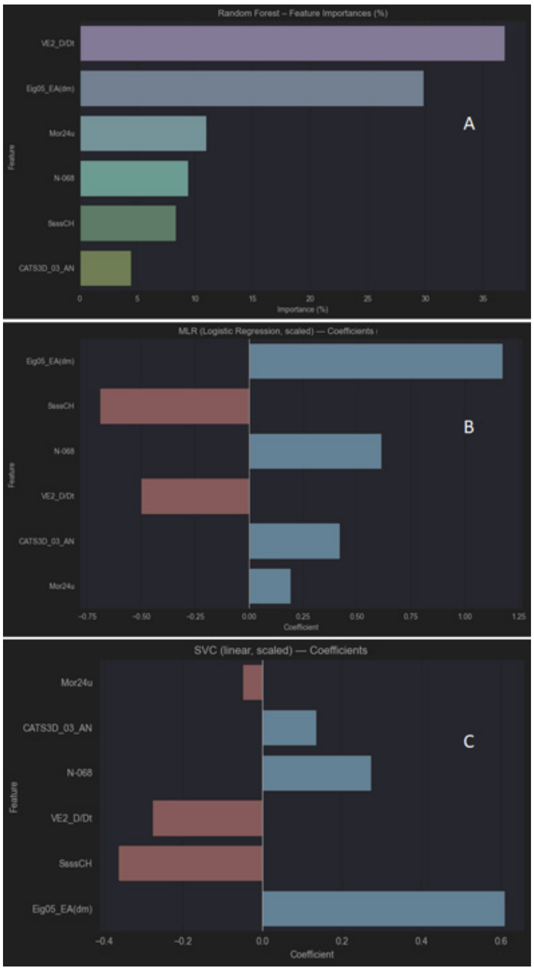


**Figure S4.** Contribution of descriptors to the random forest model (A), logistic regression (B), and support vector classifier (C) model of the AS4 system.

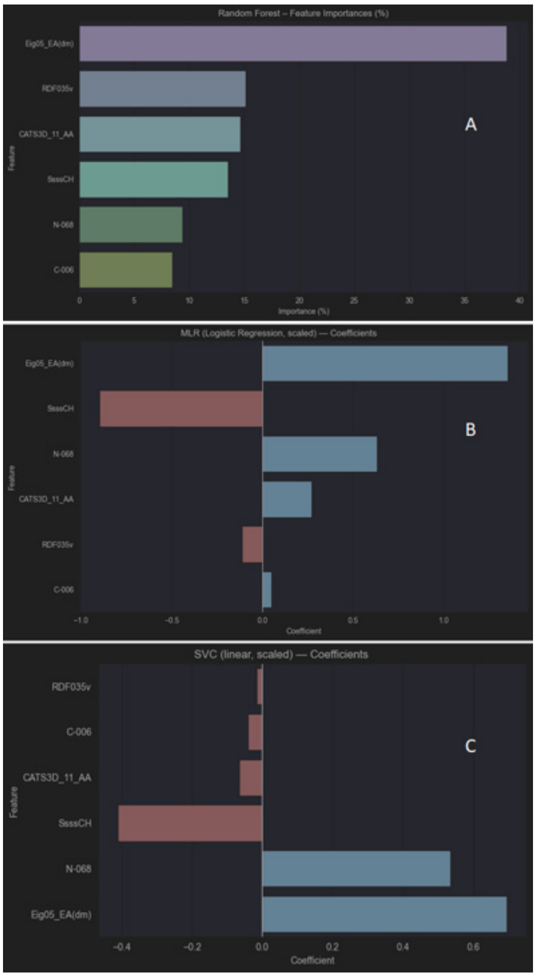


**Figure S5.** Contribution of descriptors to the random forest model (A), logistic regression (B), and support vector classifier (C) model of the A5 system.

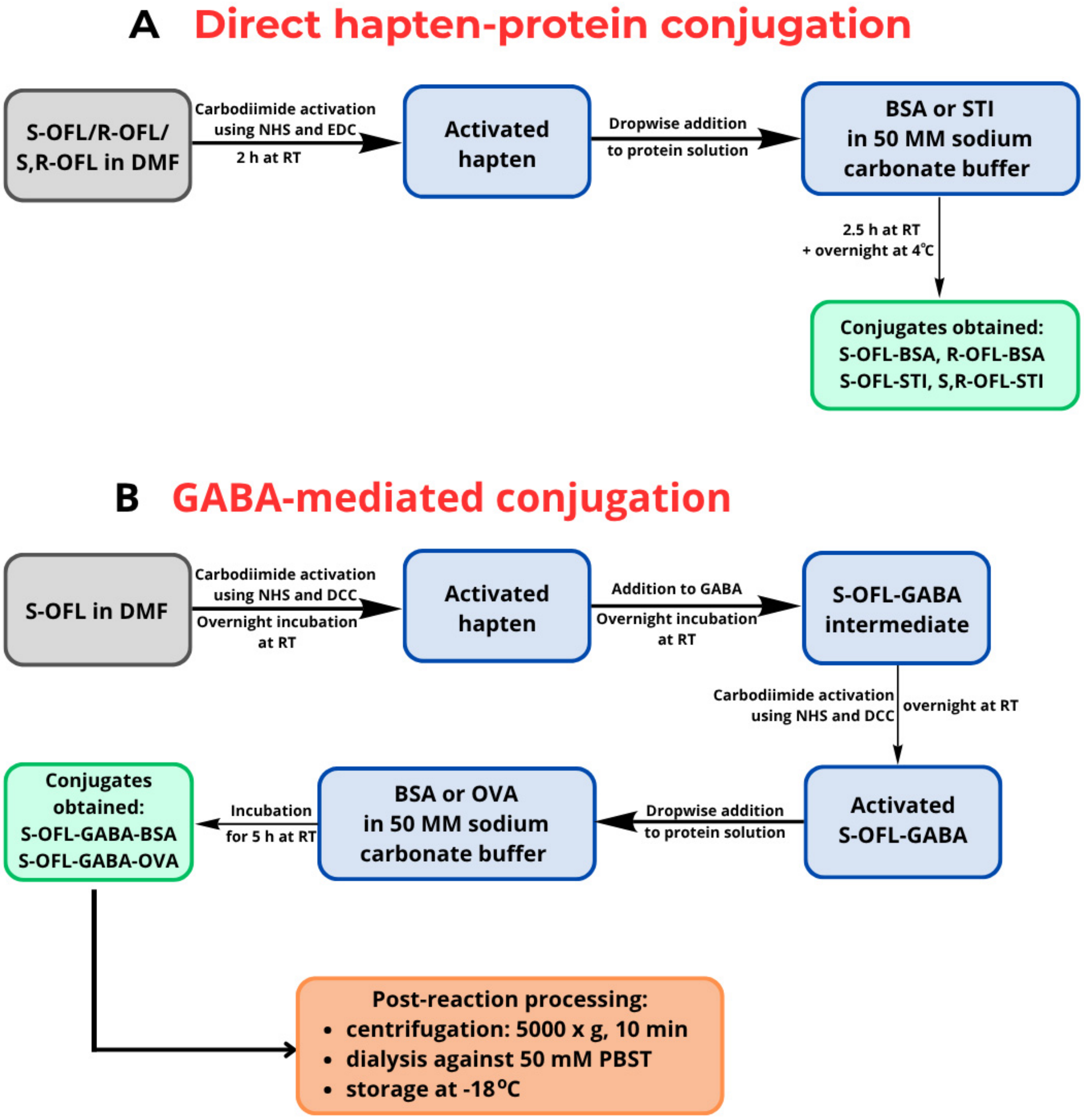


**Figure S6.** Schematic representation of the synthetic routes used for hapten–protein conjugate preparation.
